# Soluble CD13 induces inflammatory arthritis by activating the bradykinin receptor B1

**DOI:** 10.1172/JCI151827

**Published:** 2022-06-01

**Authors:** Pei-Suen Tsou, Chenyang Lu, Mikel Gurrea-Rubio, Sei Muraoka, Phillip L. Campbell, Qi Wu, Ellen N. Model, Matthew E. Lind, Sirapa Vichaikul, Megan N. Mattichak, William D. Brodie, Jonatan L. Hervoso, Sarah Ory, Camila I. Amarista, Rida Pervez, Lucas Junginger, Mustafa Ali, Gal Hodish, Morgan M. O’Mara, Jeffrey H. Ruth, Aaron M. Robida, Andrew J. Alt, Chengxin Zhang, Andrew G. Urquhart, Jeffrey N. Lawton, Kevin C. Chung, Tristan Maerz, Thomas L. Saunders, Vincent E. Groppi, David A. Fox, M. Asif Amin

**Affiliations:** 1 Division of Rheumatology and Clinical Autoimmunity Center of Excellence, Department of Internal Medicine, University of Michigan, Ann Arbor, Michigan, USA.; 2 Department of Rheumatology and Immunology, West China Hospital, Sichuan University, Chengdu, Sichuan, China.; 3 Department of Orthopedic Surgery, University of Michigan Health System, Ann Arbor, Michigan, USA.; 4 Pharmacology Department,; 5 Department of Computational Medicine and Bioinformatics,; 6 Biomedical Research Core Facilities, Transgenic Animal Model Core, and; 7 Center for Discovery of New Medicine, University of Michigan, Ann Arbor, Michigan, USA.

**Keywords:** Autoimmunity, Arthritis, Chemokines, G protein&ndash;coupled receptors

## Abstract

CD13, an ectoenzyme on myeloid and stromal cells, also circulates as a shed, soluble protein (sCD13) with powerful chemoattractant, angiogenic, and arthritogenic properties, which require engagement of a G protein–coupled receptor (GPCR). Here we identify the GPCR that mediates sCD13 arthritogenic actions as the bradykinin receptor B1 (B1R). Immunofluorescence and immunoblotting verified high expression of B1R in rheumatoid arthritis (RA) synovial tissue and fibroblast-like synoviocytes (FLSs), and demonstrated binding of sCD13 to B1R. Chemotaxis, and phosphorylation of Erk1/2, induced by sCD13, were inhibited by B1R antagonists. In ex vivo RA synovial tissue organ cultures, a B1R antagonist reduced secretion of inflammatory cytokines. Several mouse arthritis models, including serum transfer, antigen-induced, and local innate immune stimulation arthritis models, were attenuated in *Cd13^–/–^* and *B1R^–/–^* mice and were alleviated by B1R antagonism. These results establish a CD13/B1R axis in the pathogenesis of inflammatory arthritis and identify B1R as a compelling therapeutic target in RA and potentially other inflammatory diseases.

## Graphical abstract

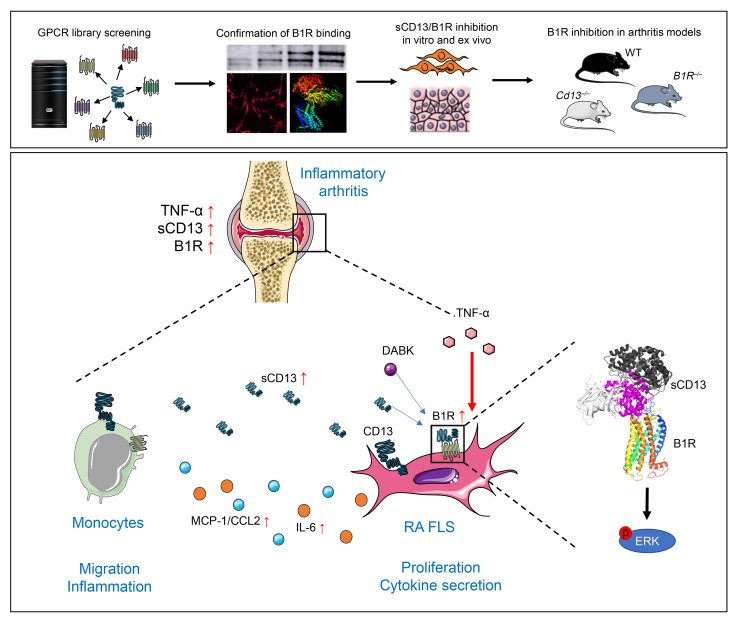

## Introduction

Rheumatoid arthritis (RA) is an autoimmune disease characterized by infiltration of monocytes (MNs) and T cells into synovial tissue (ST), accompanied by angiogenesis and hyperplasia of fibroblast-like synoviocytes (FLSs) (1–3). Angiogenesis contributes to ST proliferation, pannus development, and leukocyte ingress. Aminopeptidase N (CD13; EC 3.4.11.2) is a transmembrane, cytokine-inducible ectopeptidase, expressed by RA FLSs, MNs, and endothelial cells (ECs) (4–6). Soluble CD13 (sCD13), shed from the FLS surface by the protease MMP-14, is found at much higher levels in RA synovial fluids compared with osteoarthritis or normal serum, and exerts powerful chemotactic effects at a concentration equal to the gradient between RA synovial fluid and serum concentrations. sCD13 contributes to both T cell and MN migration in RA, as depletion of sCD13 from RA synovial fluids substantially reduces the ability of such fluids to trigger MN or T cell chemotaxis (6–8). Moreover, sCD13 induces FLS proliferation, angiogenesis, and acute arthritis when injected into mouse knees (6–8). All these effects of sCD13 are independent of its enzymatic activity (6–8).

In other studies, CD13 contributed to MN recruitment in an acute inflammatory model of peritonitis (5, 9) and to migration, phagocytosis, and angiogenesis of various cell types (10, 11). However, these investigations did not distinguish the roles of membrane-anchored CD13 versus sCD13. Our recent work showed that sCD13 induces migration of cytokine-activated T cells (Tcks) and MNs by binding to G protein–coupled receptors (GPCRs) (6, 8).

The bradykinin receptor B1 (B1R) is a GPCR that is inducible under inflammatory conditions and has important functions in inflammation (12, 13). B1R blockade attenuated IL-1β–induced leukocyte-EC interactions and leukocyte emigration (12, 13). B1R activation induces increased rolling, adhesion, and migration of polymorphonuclear leukocytes, and B1R antagonism reduced IL-1β–induced leukocyte accumulation (13, 14). Several steps in leukocyte recruitment, such as rolling, adherence, and emigration, are reduced in B1R-knockout (*B1R^–/–^*) mice (15, 16). Mice deficient in both B1R and B2R are resistant to the development of collagen antibody–induced arthritis (CAIA) and have less IL-1β and IL-6 in joint homogenates (17). However, the CAIA model induces acute arthritis that involves formation of immune complexes and activation of complement that may be relatively independent of sCD13.

Here we determined the role of sCD13 in arthritis development and identified that B1R is a receptor for sCD13, through screening of a GPCR expression library. We confirmed the binding of sCD13 to B1R by immunofluorescence and immunoblotting. We examined sCD13-mediated MN migration and phosphorylation of signaling molecules, with or without B1R inhibitors (SSR240612 or R715). RA ST organ culture assays, which use freshly procured RA ST from patients with established RA, were also used to measure the secretion of cytokines in the presence or absence of a B1R antagonist. Animal models of arthritis, including acute inflammatory models and an antigen-induced arthritis model, were performed, using wild-type (WT), Cd13-knockout (*Cd13^–/–^*), and *B1R^–/–^* mice, with or without B1R antagonists. Since sCD13/B1R influences most cell types involved in RA, and inhibition of this interaction shows potent efficacy in ex vivo models of RA and animal models of arthritis, these results point to central roles for sCD13 and B1R in joint inflammation, and suggest that targeting sCD13 or its receptor could be a useful approach to the treatment of inflammatory diseases such as RA.

## Results

### Cd13^–/–^ mice develop significantly less inflammatory arthritis when injected with TNF-α.

**Figure 1:**
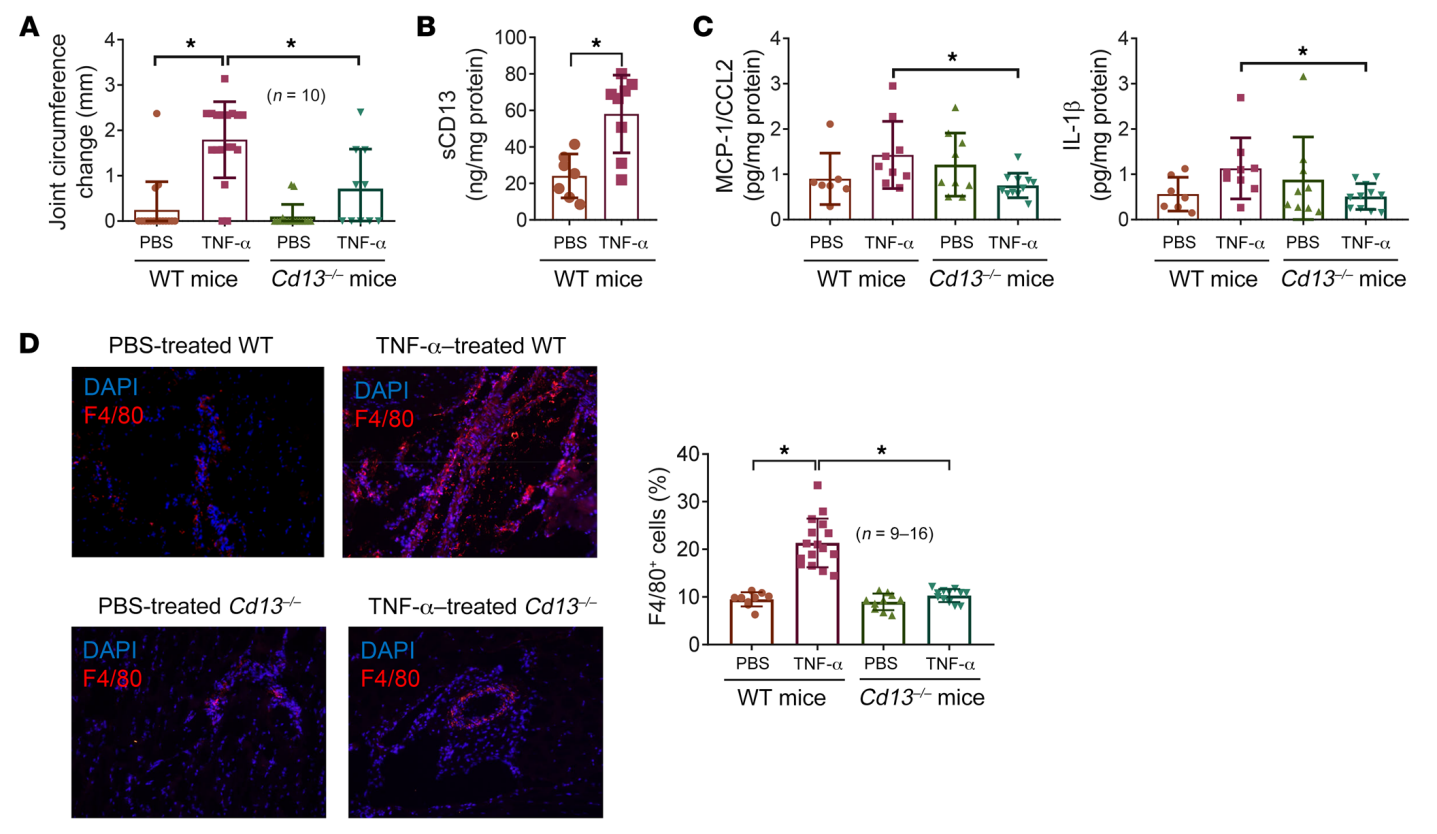
Cd13-knockout (*Cd13^–/–^*) mice develop less inflammation compared with WT mice when injected with TNF-α. TNF-α was injected into *Cd13^–/–^* and C57BL/6J WT mouse knee joints. PBS served as a negative control. (**A**) *Cd13^–/–^* mice developed significantly less knee swelling when TNF-α was injected into their knees compared with WT mice. *n* = number of knee joints per group: WT-PBS, *n* = 16; WT–TNF-α, *n* = 18; *Cd13^–/–^*-PBS, *n* = 16; *Cd13^–/–^*–TNF-α, *n* = 10. (**B**) sCD13 was significantly elevated in TNF-α–injected knees (*n* = 8) in WT mice compared with PBS-injected mice (*n* = 7). (**C**) Knee homogenates from TNF-α–treated WT mice (*n* = 9) showed significantly higher MCP-1/CCL2 and IL-1β compared with TNF-α–treated *Cd13^–/–^* mice (*n* = 11). *n* = number of mouse knees per group. (**D**) TNF-α–treated WT mouse cryosections showed a marked increase in F4/80 staining compared with sections from PBS-treated mice, and this was not observed in the TNF-α–treated *Cd13^–/–^* mice. Red staining represents F4/80 for MNs/macrophages, while blue (DAPI) stains nucleus. Original magnification, ×200. Results are expressed as mean ± SD. *P < 0.05. Significance was determined by Kruskal-Wallis test (**A** and **C**), unpaired, 2-tailed Student’s *t* test (**B**), and 1-way ANOVA (**C** and **D**).

To test the role of CD13 in the induction of acute arthritis by a proinflammatory cytokine, we injected TNF-α (14 μM) into *Cd13^–/–^* and WT mouse knees (*n* = 10 knees per group). After 24 hours, WT mouse joints injected with TNF-α had a significantly greater increase in joint swelling compared with *Cd13^–/–^* mouse knee joints injected with TNF-α (*P* < 0.05) or WT joints injected with PBS (*P* < 0.05) (Figure 1A). In the knee homogenates, significant elevation of sCD13 after TNF-α injection was observed in WT mice compared with the PBS group (Figure 1B). In addition, levels of monocyte chemoattractant protein-1 (MCP-1/CCL2) and IL-1β in TNF-α–treated WT mice were significantly higher than those in TNF-α–treated *Cd13^–/–^* mice (Figure 1C). Similar results were observed with other cytokines, including IFN-γ (0.10 ± 0.04 vs. 0.06 ± 0.02 pg/mg protein, WT–TNF-α vs. *Cd13^–/–^*–TNF-α, *P* < 0.05), IL-1α (7.87 ± 2.14 vs. 2.69 ± 0.85 pg/mg protein, WT–TNF-α vs. *Cd13^–/–^*–TNF-α, *P* < 0.05), and TNF-α (0.24 ± 0.09 vs. 0.09 ± 0.04 pg/mg protein, WT–TNF-α vs. *Cd13^–/–^*–TNF-α, *P* < 0.05). This was also accompanied by significantly more MN/macrophage infiltration in WT mice compared with *Cd13^–/–^* mice (Figure 1D). These results imply that CD13 can act downstream of TNF-α in the induction of joint inflammation.

### Identification of the GPCR activated by sCD13 as B1R.

As we previously showed that sCD13 acts through a GPCR, we sought to identify a receptor for sCD13 that could mediate its arthritogenic effects (6, 8). Using a previously described assay, a library of human GPCRs was screened with sCD13 as a ligand/probe (18). A strong signal was detected from the B1R-expressing clone. Subsequent experiments were therefore performed to further validate the role of B1R as a sCD13 receptor and to investigate the functional importance of this receptor/ligand interaction.

### sCD13 directly interacts with B1R.

**Figure 2:**
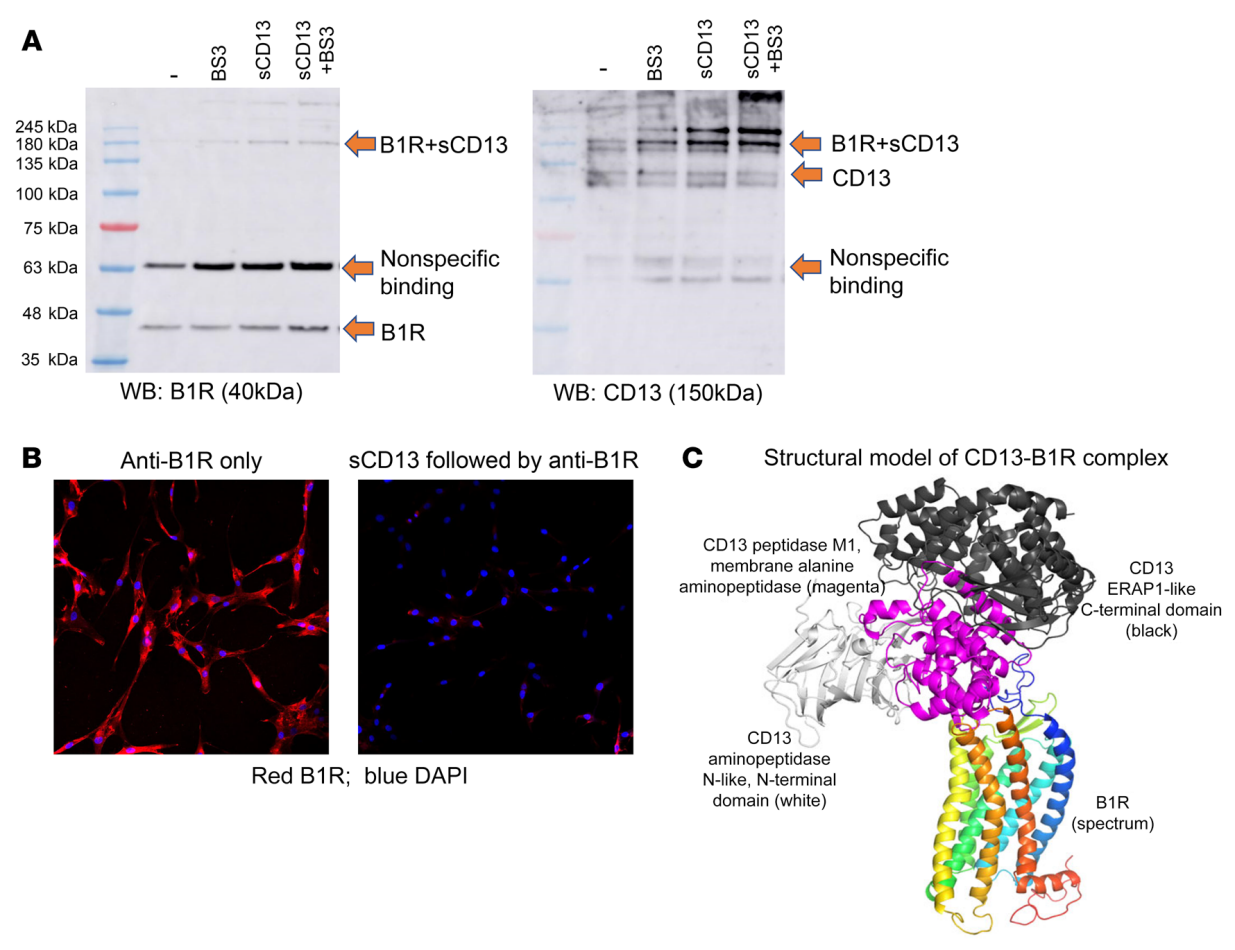
sCD13 is a new ligand for B1R. (**A**) Western blot of RA FLSs preincubated with sCD13 with or without addition of a BS3 cross-linker. A 40 kDa protein band was identified as the B1R monomer (labeled as B1R), and a 180 kDa band (labeled as B1R+sCD13), corresponding to the sum of the molecular masses of sCD13 plus B1R, was identified by both B1R and CD13 antibodies. (**B**) Immunofluorescence was performed to detect B1R on RA FLSs. RA FLSs were stained with an anti-B1R antibody (red), and nuclei were stained by DAPI (blue). Preincubation of RA FLSs with sCD13 blocked the binding of anti-B1R antibody to B1R (right panel). Original magnification, ×200. Experiments were performed twice. (**C**) C-I-TASSER program was used to predict the protein structure of monomeric B1R and CD13. Structure of the complex between B1R (colored in spectrum, with blue to red for N- to C-terminal) and CD13 (white, magenta, and black for its N-terminal, center, and C-terminal domains, respectively) is shown.

To examine the binding of sCD13 to B1R, cell lysates were prepared from RA FLSs, and Western blots were performed. In lysates of FLS membranes, we identified the B1R 40 kDa protein band (Figure 2A) as the B1R monomer. After stripping these blots, we probed it with an antibody that detects CD13 and found a 180 kDa band that corresponds to the sum of the molecular masses of sCD13 plus B1R and represents a B1R-sCD13 complex, indicating binding of sCD13 to B1R. Comparing the left and right panels, both anti-B1R and anti-CD13 antibodies detected this band (Figure 2A). The cross-linking agent BS3 enhanced the intensity of this complex, but was not required for its formation and detection.

B1R was highly expressed in RA FLSs as determined by flow cytometry (Supplemental Figure 1; supplemental material available online with this article; https://doi.org/10.1172/JCI151827DS1). Immunofluorescence was then performed to determine whether preincubation of RA FLSs with sCD13 prevents or reduces the binding of anti-B1R antibody to B1R. Preincubation of RA FLSs with sCD13 blocked the binding of anti-B1R antibody to B1R, further confirming that sCD13 is a new ligand for B1R (Figure 2B).

As shown in Figure 2C and Supplemental Table 1, the CD13-B1R complex structural model suggests that the interaction mainly involves the CD13 center domain (CD13 peptidase M1) and the B1R extracellular side (the top part of the transmembrane helix bundle). Meanwhile, the N- and C-terminal domains of CD13 do not seem to contribute residues to the contacts with B1R.

### TNF-α and IL-1β increase B1R mRNA and protein expression in FLSs.

**Figure 3:**
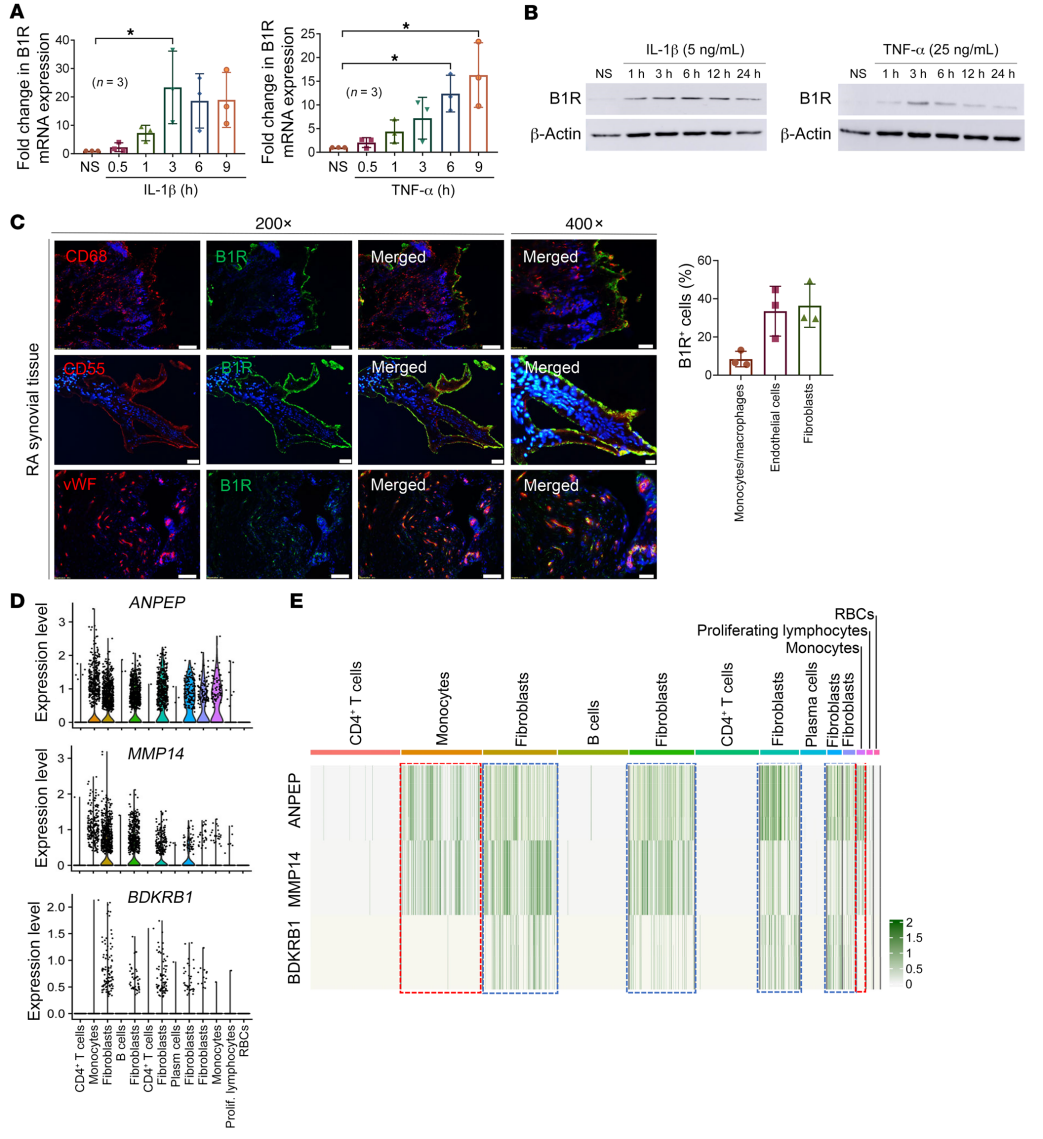
B1R is expressed in RA FLSs and ST. (**A** and **B**) IL-1β and TNF-α induced B1R mRNA and protein expression in RA FLSs in a time-dependent manner as determined by quantitative PCR and Western blotting. *n* = 3 RA FLSs used in each group. (**C**) B1R is highly expressed in RA in vivo. Immunofluorescence staining was performed targeting B1R (green), myeloid cell marker CD68 (red), fibroblast marker CD55 (red), EC marker vWF (red), and nuclear staining DAPI (blue). Representative pictures from 3 RA patients. Quantification was done by counting of B1R-positive cells in 5 fields per patient. Original magnification, ×200 and ×400. (**D**) Analysis of single-cell RNA-Seq data from Zhang et al. (71). showed that *BDKRB1* is predominantly expressed on FLSs, while *ANPEP* and its sheddase *MMP14* are expressed on FLSs and MNs. (**E**) All FLS (blue boxes) subsets coexpress *ANPEP*, *MMP14*, and *BDKRB1*, while the 2 MN clusters (red boxes) have high levels of *ANPEP* and *MMP14* but low expression of *BKDRB1*. Results are shown as mean ± SD. *P < 0.05. Significance was determined by Kruskal-Wallis test or 1-way ANOVA (**A**).

To determine whether B1R is inducible at mRNA and protein levels, RA FLSs were stimulated with TNF-α or IL-1β. We found that B1R mRNA was inducible in RA FLSs. IL-1β and TNF-α increased B1R mRNA expression (Figure 3A) with the peak increase between 3 and 6 hours. Next, Western blots were performed with cell lysates from cytokine-stimulated FLSs, which demonstrated a marked increase in B1R protein level in cytokine-stimulated FLSs (Figure 3B).

### B1R is highly expressed in RA ST.

To confirm that B1R is expressed in RA ST, we performed immunofluorescence with RA ST cryosections. B1R was highly expressed in RA ST, suggesting a potential role for B1R in human RA (Figure 3C and Supplemental Figure 2). To determine the cell types in the RA synovium that express B1R, we costained the RA tissue with markers for fibroblasts (CD55 or CD90), MNs/macrophages (CD68), and ECs (vWF). We found that B1R was expressed on RA FLSs, ECs, and MNs/macrophages.

To determine the expression pattern of B1R, CD13, and its primary sheddase MMP-14 on various cell types in RA synovium, we analyzed published single-cell RNA-Seq results generated from 27 RA synovia with a total of 7487 flow cytometry–sorted fibroblasts, MNs, T cells, and B cells. A total of 13 clusters including 5 fibroblast subsets (clusters 2, 4, 6, 8, and 9), 2 MN subsets (clusters 1 and 10), and other immune cells were identified (Supplemental Figure 3). From the violin plots and the uniform manifold approximation and projection (UMAP) analyses (Figure 3D and Supplemental Figure 3), it appears that *BDKRB1* (encoding B1R) was predominantly expressed on FLSs, while *ANPEP* (encoding CD13) and its sheddase *MMP14* were expressed on FLSs and MNs. Examining the expression in different cell clusters in detail, we found that all FLS subsets (blue boxes) coexpressed *ANPEP*, *MMP14*, and *BDKRB1*, while the two MN clusters (red boxes) had high levels of *ANPEP* and *MMP14* but low expression of *BDKRB1* (Figure 3E). The discrepancy between tissue staining and the RNA-Seq results of B1R expression in RA tissues could result from limited read depth of RNA-Seq, as well as the loss of transient B1R expression during transport and handling of tissues before sequencing. These results suggest that CD13 can be potentially shed by MMP-14 from FLSs and MNs, and that the effect of sCD13 can act in an autocrine or paracrine fashion on B1R in FLSs and to a lesser extent in other cell types.

### sCD13 induces MN migration via B1R.

**Figure 4:**
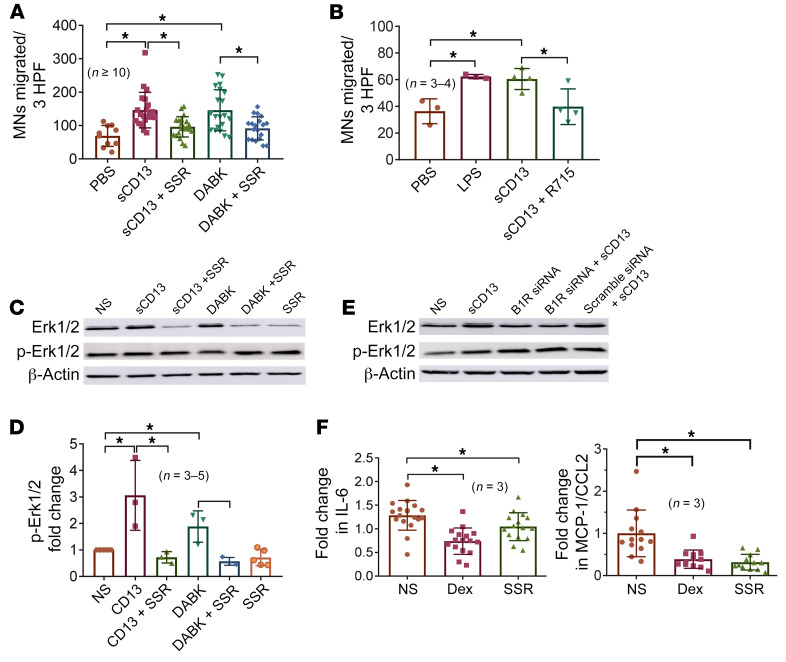
B1R antagonist inhibits sCD13-mediated MN migration, FLS signaling, and cytokine production. (**A**) SSR240612 (*n* = 21) significantly inhibited sCD13-induced MN migration (*n* = 21). DABK-induced MN migration (*n* = 20) was also inhibited by SSR240612 (*n* = 20), suggesting that DABK and sCD13 are 2 ligands of B1R. PBS (*n* = 10) was used as a negative control. *n* = number of wells. HPF, high-power fields. (**B**) Another B1R inhibitor, R715 (*n* = 4), also significantly inhibited sCD13-induced MN migration (*n* = 4). Lipopolysaccharide (LPS, *n* = 3) and PBS (*n* = 3) were used as positive and negative controls, respectively. *n* = number of assays. (**C**) sCD13- or DABK-stimulated phospho-Erk1/2 was significantly reduced by SSR240612 in RA FLSs from 4 different patients. (**D**) Quantification of Western blots. *n* = number of blots. (**E**) RA FLSs transfected with a B1R-silencing construct showed that sCD13-stimulated Erk1/2 phosphorylation was markedly decreased in comparison with scrambled RNA. (**F**) B1R inhibitor SSR240612 inhibited cytokines MCP-1/CCL2 and IL-6 in RA ST. Dexamethasone was used as a control for cytokine inhibition. *n* = number of replicates from 3 RA patients. Results represent the mean ± SD. *P < 0.05. Significance was determined by Kruskal-Wallis test (**A**) and 1-way ANOVA (**B**, **D**, and **F**). SSR, SSR240612; NS, nonstimulated; Dex, dexamethasone.

To evaluate the role of B1R in MN migration mediated by sCD13 and des-Arg9-bradykinin (DABK), we performed chemotaxis assays using normal human MNs in modified Boyden chambers as previously described (19, 20). sCD13-induced MN migration was inhibited by SSR240612 (a B1R inhibitor), as was DABK-induced MN migration, suggesting that DABK and sCD13, the 2 ligands of B1R, show similar functional effects. R715, another B1R inhibitor, also reduced sCD13-induced MN migration, although the inhibition with SSR240612 was more robust than that with R715 (Figure 4, A and B).

### B1R antagonists inhibit sCD13- and DABK-induced phosphorylation of Erk1/2 in RA FLSs.

To assess the role of B1R in phosphorylation of Erk1/2, RA FLSs were preincubated with B1R inhibitor (SSR240612) for 1 hour before stimulation with sCD13 (4.8 nM) for 15–20 minutes, and Western blots were performed. We found that sCD13-stimulated phosphorylation of Erk1/2 was markedly reduced by SSR240612 in RA FLSs. This assay was repeated with FLSs harvested from 4 different RA patients. We also used the bradykinin agonist DABK to stimulate FLSs with or without a B1R antagonist. DABK-induced phosphorylation of Erk1/2 was reduced by the B1R antagonist (SSR240612), suggesting that both sCD13 and DABK are ligands for B1R on FLSs. Western blots in Figure 4, C and D, and Supplemental Figure 4 show the effects of both ligands of B1R used for an identical duration in the same RA FLS line. We also confirmed our data by transfecting RA FLSs with B1R siRNA (Supplemental Figure 5) and found that RA FLSs with B1R silencing exhibited decreased sCD13-stimulated Erk1/2 phosphorylation compared with FLSs transfected with scrambled RNA (Figure 4E). Therefore, B1R mediates the signal transduction induced by sCD13.

### B1R antagonist inhibits secretion of proinflammatory cytokines from RA ST organ cultures.

We have recently published that sCD13 exerts proinflammatory effects on multiple cell types that play important roles in RA ST growth. Moreover, neutralizing antibody against CD13 inhibited the secretion of proinflammatory cytokines by RA ST organ cultures ex vivo (8, 21). We therefore evaluated the contribution of B1R in RA ST secretion of inflammatory cytokines by performing ex vivo assays in which ST obtained from RA patients was cultured with a B1R antagonist (SSR240612, 50 μM).

RA ST samples cultured in medium containing SSR240612 showed significantly decreased levels of MCP-1/CCL2 and IL-6 in the supernatants by ELISA (Figure 4F). The degree of inhibition of production of these mediators was similar to the effect of dexamethasone, used in this assay as a positive control.

### A B1R antagonist ameliorates zymosan-induced arthritis in WT mice.

**Figure 5:**
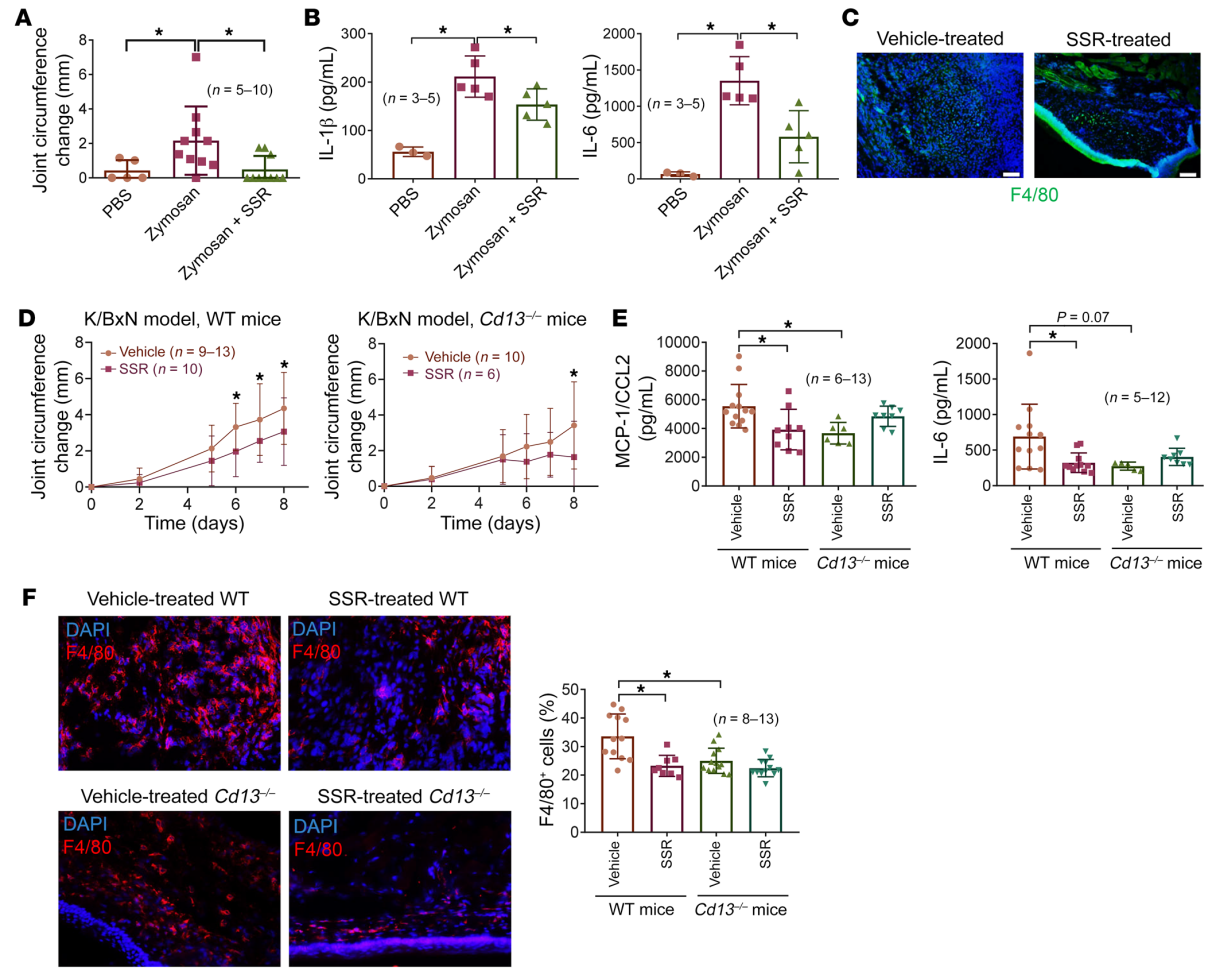
Blockade of B1R attenuates ZIA and K/BxN serum transfer arthritis in mice. (**A**) B1R antagonist SSR240612 significantly reduced ZIA in WT mice compared with vehicle-treated mice. *n* = number of mice per group: PBS, *n* = 5; zymosan, *n* = 10; zymosan + SSR, *n* = 10. (**B**) Knee homogenates from SSR240612-treated mice showed significantly lower IL-1β and IL-6 in comparison with vehicle-treated mice. *n* = number of mouse knees per group: PBS, *n* = 3; zymosan, *n* = 5; zymosan + SSR, *n* = 5. (**C**) Immunofluorescence of knees from SSR240612-treated mice shows a marked decrease in macrophage marker F4/80 (green). Representative sections from each group are shown. Original magnification, ×200. (**D**) Vehicle-treated WT mice (*n* = 9–13) had profound serum transfer arthritis as measured by joint circumference compared with SSR240612-treated WT mice (*n* = 10). There was no difference in arthritis between SSR240612-treated *Cd13^–/–^* mice (*n* = 6) and vehicle-treated *Cd13^–/–^* mice (*n* = 10) except for day 8. (**E**) Significant decrease in MCP-1/CCL2 and IL-6 in ankle homogenates from SSR240612-treated WT (*n* = 9) compared with vehicle-treated WT mice (*n* = 13) is shown. In contrast, significant increase in the cytokines was observed in the SSR240612-treated *Cd13^–/–^* mice (*n* = 8) compared with vehicle-treated *Cd13^–/–^* mice (*n* = 6). (**F**) SSR240612-treated WT and *Cd13^–/–^* mouse cryosections showed a marked decrease in F4/80 staining compared with sections from vehicle-treated mice. Red staining represents F4/80 for MNs/macrophages, while blue (DAPI) stains nucleus. Original magnification, ×200. Results are expressed as mean ± SD. *P < 0.05. Significance was determined by Kruskal-Wallis test (**A** and **E**), 1-way ANOVA (**B** and **F**), and 2-way ANOVA (**D**).

To evaluate the role of B1R in acute models of arthritis, we performed zymosan-induced arthritis (ZIA) by administering a single intra-articular injection of zymosan to WT mice, followed by observation of these mice over a 48-hour period. We found that during this time frame, the levels of sCD13 in the knee joints were significantly elevated in the zymosan-injected mice compared with PBS controls (Supplemental Figure 6). These mice were also injected with a B1R antagonist on day 0 and after 24 hours. WT mice treated with SSR240612 showed a significant decrease in knee swelling 48 hours after ZIA induction (Figure 5A). We also measured proinflammatory cytokines in homogenates from knees of mice treated with SSR240612 or vehicle. Mice treated with SSR240612 produced less IL-6 and IL-1β compared with vehicle-treated mice, suggesting an important role for B1R and its ligands in acute inflammatory arthritis (Figure 5B). Cryosections of knees from mice treated with the B1R antagonist exhibited a marked decrease in MN recruitment compared with the vehicle-treated group (Figure 5C).

### Roles of B1R and sCD13 in K/BxN serum–induced arthritis.

To determine the role of sCD13 and B1R in an antibody-driven animal model of arthritis, we induced K/BxN serum transfer arthritis in WT and *Cd13^–/–^* mice as previously described (22, 23). WT mice treated with vehicle developed severe arthritis starting from day 5, which was significantly reduced by the B1R inhibitor SSR240612 given daily at 0.82 mM by i.p. injection (Figure 5D). These mice were euthanized on day 8, and ankles were harvested for cryosectioning and homogenization. We found that MCP-1/CCL2 and IL-6 were significantly lower in SSR240612-treated WT mouse arthritic ankle homogenates in comparison with vehicle-treated WT mice, suggesting that B1R plays an important role in the expression of these cytokines in K/BxN serum–induced arthritis (Figure 5E). Cryosections of K/BxN serum transfer arthritic WT ankles from mice treated with the B1R antagonist exhibited a marked decrease in MN/macrophage recruitment compared with the vehicle-treated group as defined by staining for F4/80 (Figure 5F).

To elucidate the role of CD13 in this model, we induced K/BxN serum transfer arthritis in *Cd13^–/–^* mice. We found that the *Cd13^–/–^* mice developed less arthritis compared with WT mice (Supplemental Figure 7A). This was accompanied by lower cytokine production and immune cell infiltration (Figure 5, E and F). To further characterize the role of B1R in this system, we treated the *Cd13^–/–^* mice with a B1R inhibitor. *Cd13^–/–^* mice treated with a B1R antagonist had less arthritis compared with vehicle-treated mice, but a significant decrease in arthritis severity in *Cd13^–/–^* mice was not observed until day 8, whereas WT mice treated with a B1R antagonist developed significantly less arthritis starting on day 6 (Figure 5D). In addition, B1R antagonism was able to further decrease arthritis in *Cd13^–/–^* mice compared with WT mice (Supplemental Figure 7B). These results also indicate that a B1R antagonist can have anti-arthritic effects beyond inhibiting the actions of sCD13, likely by blocking activation of B1R by bradykinins.

Paradoxically, the cytokines in joints of *Cd13^–/–^* mice treated with a B1R inhibitor were elevated slightly (Figure 5E). The reason for this is unknown. It might be due to a compensatory mechanism during the development of *Cd13^–/–^* mice that is unmasked upon blockade of B1R. This result does suggest that the anti-arthritic effects of a B1R antagonist are not entirely dependent on lowering of the levels of proinflammatory cytokines in the joints.

### Effect of B1R blockade in methylated BSA antigen–induced arthritis.

**Figure 6:**
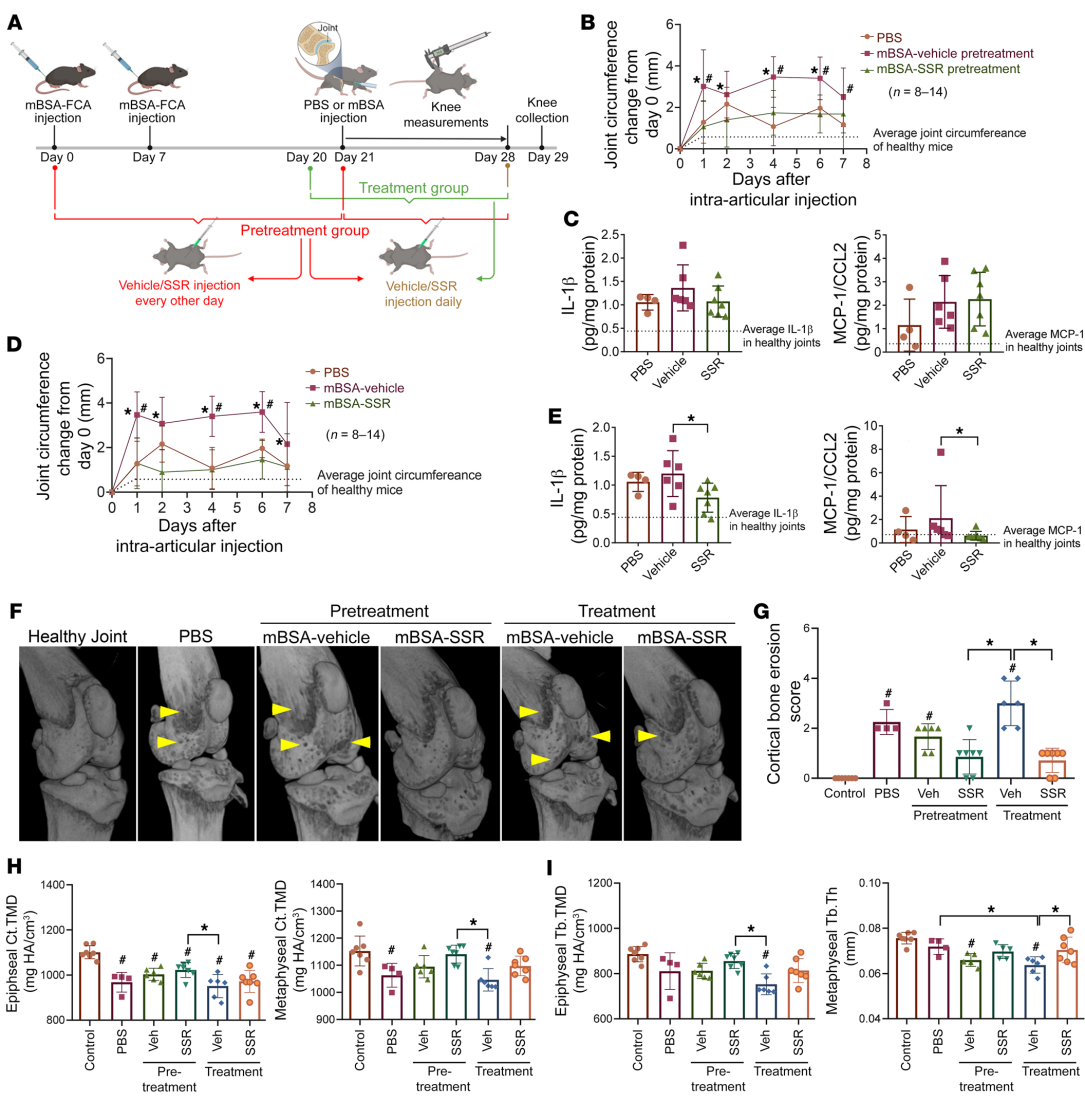
Blockade of B1R attenuates mBSA antigen–induced arthritis and bone erosion in mice. (**A**) Schematic of study design. (**B**) In the pretreatment group of animals, mBSA injection into the knee significantly induced joint swelling on days 1, 4, 6, and 7 compared with PBS, and B1R blockade significantly reduced knee circumference induced by mBSA on days 1, 2, 4, and 6. *n* = number of knees per group: PBS, *n* = 8; vehicle, *n* = 12; SSR, *n* = 14. (**C**) No significant changes in IL-1β and MCP-1/CCL2 were found in knee homogenates among groups. (**D**) In the treatment group of animals, mBSA injection into the knee significantly induced joint swelling on days 1, 4, and 6 compared with PBS, and B1R blockade significantly reduced knee circumference induced by mBSA on days 1, 2, 4, 6, and 7. *n* = number of knees per group: PBS, *n* = 8; vehicle, *n* = 12; SSR, *n* = 14. (**E**) Significant reduction in IL-1β and MCP-1/CCL2 was found in knee homogenates from SSR-treated animals compared with vehicle-treated ones. (**F**) Representative 3D μCT reconstructions of murine knee joints. Arrowheads indicate sites of cortical bone erosion. (**G**) Qualitative scoring of cortical bone erosion demonstrates amelioration of mBSA-induced cortical destruction in SSR240612-treated mice. *n* = number of knees per group: healthy control, *n* = 7; PBS *n* = 4; pretreatment vehicle, *n* = 6; pretreatment SSR, *n* = 7; treatment vehicle, *n* = 6; treatment SSR, *n* = 7. (**H** and **I**) Analysis of the epiphyseal and metaphyseal cortical tissue mineral density (Ct.TMD), trabecular tissue mineral density (Tb.TMD), and mean trabecular thickness (Tb.Th) showed catabolic bone remodeling in all mBSA-treated mice relative to healthy control, and significant inhibition of mBSA-induced cortical and trabecular bone loss in SSR240612-treated groups. HA, hydroxyapatite. Results are expressed as mean ± SD. *P < 0.05 vs. SSR, ^#^*P* < 0.05 vs. PBS (**B** and **D**). *P < 0.05, ^#^*P* < 0.05 vs. control (**E**, **G**–**I**). Significance was determined by 2-way ANOVA (**B** and **D**), Kruskal-Wallis test (**C**, **E**, and **G**), and 1-way ANOVA (**H** and **I**).

To determine the role of B1R in an animal model of antigen-induced arthritis, we induced arthritis in mice using methylated bovine serum albumin (mBSA). We used 2 different regimens for B1R inhibitor dosing, as depicted in Figure 6A. To determine the preventative effect of B1R in this model, we dosed SSR240612 or vehicle control every other day during the 21-day mBSA immunization period followed by daily injection for the following 7 days (pretreatment group). To determine the treatment effect of B1R, we used a treatment schedule in which animals received the B1R inhibitor (daily) only after day 20 (treatment group). We observed significant joint swelling in both models after intra-articular injection of mBSA on day 27, comparing the PBS and the mBSA-vehicle groups in both models (Figure 6, B and D), with maximum arthritic effect observed on days 4 and 6. B1R blockade in both dosing regimens significantly reduced the joint circumference, comparing the mBSA-vehicle and mBSA-SSR groups (Figure 6, B and D). These results were supported by the H&E staining and scoring of the knee joints (Supplemental Figure 8). As arthritis decreased on day 7 after intra-articular injection and knees were collected on day 8, we did not observe significant elevation of cytokine levels in the PBS and vehicle groups in both models (Figure 6, C and E). Interestingly, pretreatment with SSR240612 in this model did not alter cytokine levels (Figure 6C); however, SSR240612 given every day after the intra-articular mBSA injection led to significant reduction in IL-1β and MCP-1/CCL2 (Figure 6E). Similar results were observed with IL-1α (7.41 ± 6.27 vs. 2.81 ± 1.55 pg/mg protein, vehicle vs. SSR, *P* < 0.05). These results suggest that B1R inhibition shows potent anti-arthritic effects in an antigen-induced arthritis model.

To further determine the effect of B1R blockade on bone remodeling and erosion in the mBSA model, we imaged hind limbs using micro-CT (μCT) followed by evaluation of cortical and trabecular bone. Qualitative evaluation of femoral cortical bone demonstrated moderate induction of erosive lesions by intra-articular mBSA injection, and SSR240612 treatment significantly ameliorated the extent of bone erosion (Figure 6F, arrowheads). Mice treated with SSR240612 every day after the intra-articular mBSA injection (treatment group) and mice pretreated with SSR240612 during systemic mBSA induction (pretreatment group) exhibited significantly lower bone erosion scores compared with the vehicle group (Figure 6G). Mice in the PBS, vehicle/pretreatment, and vehicle/treatment groups had significantly greater bone erosion scores compared with healthy control mice (Figure 6G). Although some mild lesions were observable in the SSR/pretreatment and SSR/treatment groups, their cortical bone erosion scores did not vary significantly from those of control mice. Quantitative assessment of cortical mineral density corroborated qualitative scoring, indicating amelioration of mBSA-induced cortical destruction in both epiphyseal and metaphyseal regions in SSR240612-treated mice (Figure 6H). Evaluation of trabecular bone further confirmed findings related to cortical bone erosion, demonstrating catabolic bone remodeling in all mBSA-induced mice relative to healthy control and significant inhibition of mBSA-induced trabecular bone loss in SSR240612-treated groups (Figure 6I and Supplemental Figure 9). Taken together, these results demonstrate that inhibition of B1R signaling blocks the destructive, catabolic effects of antigen-induced arthritis in both cortical and trabecular bone.

### B1R^–/–^ mice are resistant to zymosan- or sCD13-induced arthritis.

**Figure 7:**
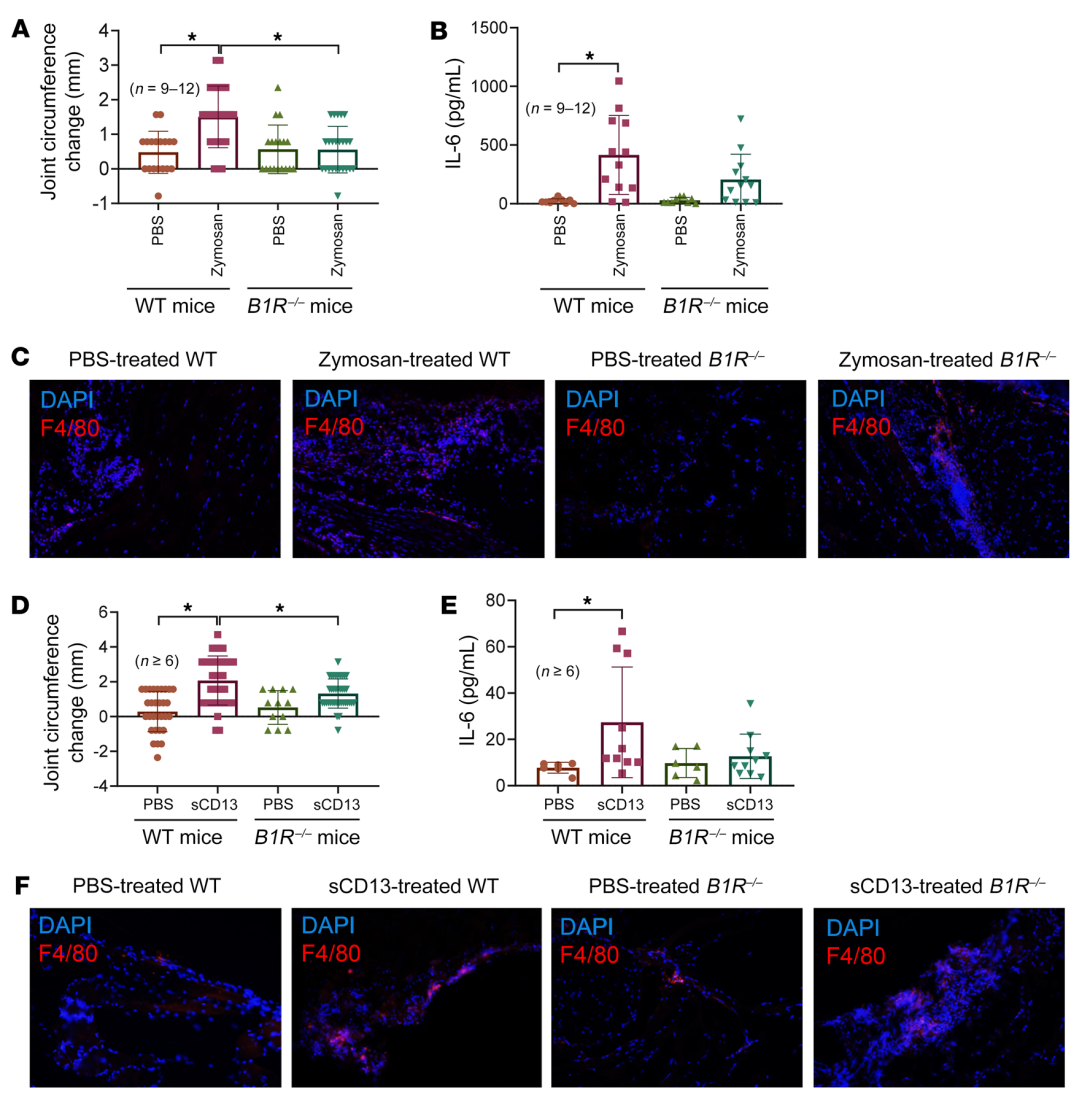
*B1R^–/–^* mice are resistant to zymosan- or sCD13-induced arthritis. (**A**) WT mice showed a significant increase in knee swelling 48 hours after ZIA induction, whereas this effect was absent in *B1R^–/–^* mice. *n* = number of mouse knees per group: WT-PBS, *n* = 18; WT-zymosan, *n* = 24; *B1R^–/–^*-WT, *n* = 18; *B1R^–/–^*-zymosan, *n* = 24. (**B**) Increase in IL-6 levels in knee homogenates in WT mice but not in *B1R^–/–^* mice after arthritis induction was observed. *n* = number of mouse knees per group: WT-PBS, *n* = 9; WT-zymosan, *n* = 12; *B1R^–/–^*-WT, *n* = 9; *B1R^–/–^*-zymosan, *n* = 12. (**C**) Zymosan-treated WT mouse cryosections showed a marked increase in F4/80 staining compared with sections from zymosan-treated *B1R^–/–^* mice. (**D**) WT mice showed a significant increase in knee swelling 48 hours after sCD13 injection, whereas this effect was absent in *B1R^–/–^* mice. *n* = number of mouse knees per group: WT-PBS, *n* = 30; WT-sCD13, *n* = 36; *B1R^–/–^*-PBS, *n* = 12; *B1R^–/–^*-sCD13, *n* = 38. (**E**) A significant increase in IL-6 in sCD13-treated joints in WT was observed compared with *B1R^–/–^* mice. *n* = number of mouse knees per group: WT-PBS, *n* = 6; WT-sCD13, *n* = 10; *B1R^–/–^*-PBS, *n* = 6; *B1R^–/–^*-sCD13, *n* = 10. (**F**) sCD13-treated WT mouse cryosections showed a marked increase in F4/80 staining compared with sections from sCD13-treated *B1R^–/–^* mice. Red staining represents F4/80 for MNs/macrophages, while blue (DAPI) stains nucleus. Results are expressed as mean ± SD. *n* = number of mice. Significance was determined by Kruskal-Wallis test (**A**, **B**, and **E**) and 1-way ANOVA (**D**). Original magnification, ×200.

To further evaluate the involvement of B1R in zymosan-induced arthritis, we induced ZIA in both WT and *B1R^–/–^* mice. WT mice showed a significant increase in knee swelling 48 hours after ZIA induction, while it was absent in *B1R^–/–^* mice (Figure 7A). We also observed a concomitant increase in the levels of many cytokines, including IL-1β, IL-6, TNF-α, and IL-1α, in knee homogenates in WT mice after arthritis induction (Figure 7B and Supplemental Table 2). Interestingly, although significant reduction in knee swelling was observed in the *B1R^–/–^* mice, we still observed elevated levels of proinflammatory cytokines, including IL-1β, IL-6, MCP-1/CCL2, IFN-γ, and TNF-α, in the joints after zymosan injection in these mice (Supplemental Table 2). Increased levels of IL-27, IFN-γ, IL-17A, and IL-10 in the zymosan-treated *B1R^–/–^* mice compared with WT mice were also observed (Supplemental Table 2). These results might suggest that actions of sCD13 that are sufficient to induce or exacerbate arthritis are not entirely dependent on inflammatory cytokine production. Certain cytokines may be more critical in this model than the others, such as IL-6 and IL-1α, as their levels were significantly elevated in zymosan-treated WT mice compared with *B1R^–/–^* mice. The antiinflammatory cytokine IL-10 was also significantly elevated in zymosan-treated *B1R^–/–^* mice compared with WT mice. We also stained for MNs/macrophages in the joints and observed increased cell infiltration in zymosan-injected WT knees, but to a lesser extent in *B1R^–/–^* mice (Figure 7C). Since we previously showed that sCD13 induced joint swelling (8), we repeated this experiment in *B1R^–/–^* mice. We found a significant reduction of joint swelling in *B1R^–/–^* mice compared with WT mice (Figure 7D). Similar to what we observed in the zymosan model, we also found an increase in IL-6 in sCD13-treated joints in WT but not in *B1R^–/–^* mice (Figure 7E). Infiltration of MNs/macrophages increased in sCD13-injected WT mice, but was not as prominent in *B1R^–/–^* mice (Figure 7F). Taken together, the results in models of acute inflammatory arthritis show that the engagement of B1R by sCD13 controls joint swelling and inflammatory cell influx, whether the process is initiated by systemic administration of autoantibodies (serum transfer arthritis) or by local stimulation of innate immune responses (by zymosan or TNF-α).

## Discussion

CD13 is a transmembrane ectopeptidase that is highly expressed by various cell types, including RA FLSs (4–6). We previously showed that sCD13 is shed from the FLS surface and plays an important role in angiogenesis, FLS proliferation, and migration of both Tcks and MNs in RA (6, 8). Depletion of sCD13 from RA synovial fluids resulted in a substantial decrease in migration of MNs or Tcks compared with sham depletion of fluids (6, 8). sCD13 also induced acute inflammation when injected into mouse knees (8). To further demonstrate the effects of sCD13 in arthritis, we generated *Cd13^–/–^* mice and found that these mice are resistant to development of acute inflammatory arthritis when injected with TNF-α into the knee joints (Figure 1).

To our knowledge, this is the first study that has identified sCD13 as a new ligand for B1R by screening of a GPCR library, and then evaluated the roles of B1R/sCD13 interactions in the pathogenesis of RA and animal models of arthritis. The prototypical ligands for B1R and B2R, kinins, consist mainly of the nonapeptide bradykinin (Arg-Pro-Pro-Gly-Phe-Ser-Pro-Phe-Arg); the decapeptide Lys-bradykinin, or kallidin; and their carboxy-terminal des-Arg9 metabolites DABK and des-Arg10-kallidin, respectively. Both bradykinin and kallidin are highly unstable peptides, have short-term effects, and are rapidly degraded by several enzymes, including angiotensin-converting enzyme, neutral endopeptidase, carboxypeptidase N, and carboxypeptidase M (24, 25). Roles for B1R and B2R have been proposed in autoimmune diseases such as RA and multiple sclerosis and inflammatory bowel diseases including Crohn’s disease and ulcerative colitis (26, 27). However, to our knowledge the connection between B1R and sCD13 in RA has never been shown before. Indeed, in this study we showed the critical involvement of the sCD13/B1R axis in various systems pertinent to RA, including in vitro, ex vivo, and in vivo models.

We found that B1R is inducible in RA FLSs by proinflammatory cytokines such as IL-1β and TNF-α at both mRNA and protein levels. B1R, once induced, does not undergo desensitization and is better equipped than B2R, the other bradykinin receptor, to mediate the development and progression of a sustained inflammatory response (28). B2R undergoes rapid agonist-induced desensitization and is unlikely to be involved in subacute and chronic inflammatory conditions (14, 28). B1R is also inducible in pathophysiologic conditions such as inflammation, trauma, burns, shock, and allergy (12–14, 29). Our data are consistent with the known inducibility of B1R in various inflammatory conditions, although prior studies did not examine B1R expression on FLSs. Flow cytometry of RA FLSs and staining of RA ST cryosections showed high expression of B1R, suggesting the importance of this receptor in RA.

To evaluate the significance of B1R in sCD13-mediated functions, several in vitro and in vivo assays were performed. The results provide strong evidence that B1R is the receptor for sCD13 that mediates most of the functions induced by sCD13. Thus, sCD13-induced MN migration was significantly inhibited by 2 inhibitors of B1R. It has been shown that B1R activation induces increased rolling, adhesion, and migration of polymorphonuclear leukocytes while B1R blockade attenuates IL-1β–induced leukocyte accumulation and leukocyte-EC interactions (12–14). *B1R^–/–^* mice develop less leukocyte recruitment rolling, adherence, and emigration (15, 16). These studies were performed using various proinflammatory stimuli that could induce sCD13, but did not employ sCD13 itself. Our data support the notion that B1R plays an important role in leukocyte recruitment and emigration, and the identification of sCD13 as a B1R ligand helps to explain these prior results. Cross-linking of membrane-bound CD13 on MNs can also lead to MN ingress (5, 30). In contrast, our recently published data and this study demonstrate that sCD13 is a potent proinflammatory factor at physiologic concentrations, and does not require cross-linking to mediate its effects.

We further assessed engagement through B1R of sCD13-induced signaling molecules. The results reinforce our previous data suggesting that sCD13 has a direct effect in increasing the phosphorylation of signaling molecules such as Erk1/2 in RA FLSs. Roles of DABK and B1R are well described in phosphorylation of mitogen-activated protein kinase (MAPK) (31, 32). In our current work, a B1R antagonist significantly inhibited both sCD13-induced and DABK-induced phosphorylation of signaling molecules, further verifying that B1R has more than one ligand. In RA FLSs transfected with B1R-silencing RNA, sCD13-stimulated Erk1/2 phosphorylation was markedly decreased in comparison with scrambled RNA, thus confirming that B1R is a receptor for sCD13.

Interactions among various cells contribute to the growth and persistence of inflammation in RA ST, including FLSs, MNs/macrophages, lymphocytes, mast cells, ECs, osteoclasts, and chondrocytes, by secretion of inflammatory and angiogenic mediators (33). The concentration of sCD13 is significantly higher in RA synovial fluids compared with serum (6, 8). Neutralization of CD13 by WM15, a monoclonal antibody specific for CD13, significantly inhibited the secretion of key proinflammatory mediators, such as MCP-1/CCL2, IL-6, and IL-8/CXCL8, in an RA ST organ culture ex vivo assay (6, 8). This suggests that sCD13 is a critical factor that drives the inflammatory response in RA by increasing cytokine secretion in RA in vivo. Here we confirmed the role of sCD13 and its receptor in secretion of proinflammatory mediators in RA by performing an RA ST organ culture ex vivo assay using a B1R antagonist. A B1R antagonist significantly reduced secretion of MCP-1/CCL2 and IL-6 in this assay. Thus both sCD13 and its receptor B1R are required for secretion of inflammatory mediators in RA, and neutralization of either blocks release of these mediators by RA ST. Our data are consistent with reports suggesting that B1R activation plays an important role in the secretion of IL-6, IL-8, IL-4, and VEGF in various cell types, but all of these studies assessed the role of B1R activation in response to its only previously known ligand, DABK (34–36).

In this study, we used various animal models of arthritis, including acute inflammatory models, a serum transfer model, and antigen-induced arthritis. Models of acute arthritis that involve intra-articular injection of arthritogenic stimuli are highly informative concerning recruitment of inflammatory cells in WT or knockout mice in vivo. Studies in the CAIA model did not find differences in MN recruitment and arthritis development when *Cd13^–/–^* mice were compared with WT mice (37). The CAIA model is an acute form of arthritis driven by formation of immune complexes and complement activation, employing inflammatory pathways that may be relatively independent of sCD13, compared with the models that we used. Joint inflammation in ZIA is mainly mediated by activation of the innate immune system, and TLR2 has been implicated to play a role (38). The K/BxN model, driven by autoantibodies against glucose-6-phosphate isomerase, is robust and replicates many features of chronic RA in humans, such as synovial hypertrophy, infiltration of MNs/macrophages, pannus invasion, and bone resorption (39, 40). The antigen-induced arthritis model is a lymphocyte-dependent experimental arthritis model that induces an acute inflammatory response followed by chronic arthritis with synovial hyperplasia, cell infiltration, and cartilage/bone erosion. We showed that the various interventions inhibiting the sCD13/B1R axis in these animal models displayed prominent anti-arthritic and joint protection effects, suggesting that sCD13 is involved in all aspects of arthritic induction.

Cytokine expression and MN ingress are 2 essential components in the progression of RA and other forms of inflammatory arthritis. We used the K/BxN serum transfer arthritis model in WT mice and *Cd13^–/–^* mice, as MN ingress is important in this model (40, 41). K/BxN arthritis was ameliorated in WT mice treated with a B1R antagonist compared with vehicle-treated WT mice. *Cd13^–/–^* mice treated with a B1R antagonist exhibited a reduction in arthritis development compared with WT mice, but a significant decrease in arthritis did not appear until day 8, suggesting the importance of both ligands of B1R in arthritis development in this model, bradykinin early and sCD13 later. Other animal models of arthritis were also employed to evaluate the effects of sCD13/B1R in arthritis development using WT, *CD13^–/–^* mice, and *B1R^–/–^* mice. ZIA, an acute model of arthritis, was performed in WT mice. WT mouse knees injected with zymosan exhibited a significant increase in circumference compared with mouse knees injected with PBS. WT mice treated with SSR240612 showed decreases in knee circumference, cytokine production, and MN infiltration compared with vehicle-treated mice. We also performed the ZIA model in *B1R^–/–^* mice, and showed that zymosan failed to induce joint swelling in these mice compared with WT mice, further supporting the essential role of B1R in this model. Intriguingly, in contrast to the SSR-treated WT mice, we observed significant increases in many cytokines in joint tissue after zymosan treatment in the *B1R^–/–^* mice. We speculate that the absence of B1R could lead to compensatory mechanisms that result in enhanced cytokine production in some situations. Indeed, we observed several cytokines to be significantly elevated in the PBS group in the *B1R^–/–^* mice. In addition, it is possible that sCD13, by facilitating MN infiltration and EC angiogenesis in the early phase of arthritis, plays a more critical role in early arthritis development, while cytokines, which are released in large quantities from FLSs, are more critical in maintaining the disease process rather than initiating it. Our results also suggest that certain cytokines, such as IL-6, IL-10, and IL-1α, might be more critical than other cytokines in this model. Effects on expression of cytokine receptors and on utilization of cytokines in B1R-deficient mice are also possible. Notwithstanding some unanswered questions about its role in cytokine production in acute arthritis models, the results in RA ST organ cultures indicate that the sCD13/B1R interaction is a critical inducer of proinflammatory and angiogenic cytokine production in chronic, established RA.

Controversies exist about the role of B1R and B2R in arthritis development in animal models. Some reports suggest that B2R has a dominant role in animal models of arthritis such as rat adjuvant-induced arthritis and peptidoglycan-polysaccharide–induced arthritis in the rat (42, 43). However, other reports indicate that B1R is a better target in animal models of arthritis while B2R deficiency did not attenuate arthritis development (17, 44). The role of B1R is also very well established in leukocyte emigration, rolling, adhesion, and inflammation (12–16). Indeed, we found that B1R blockade effectively inhibited arthritis in the mBSA antigen–induced arthritis model. The identification here of sCD13 as a B1R agonist provides a new context for understanding B1R function in inflammatory diseases.

In this study, we showed that sCD13, acting through B1R, is emerging as a potent, pleiotropic mediator in the pathogenesis of inflammatory arthritis. B1R appears to mediate all of the proinflammatory effects of sCD13, notably including the secretion of inflammatory cytokines from ex vivo RA ST organ cultures. Considering the RA ST organ culture as an established RA disease model, besides B1R blockade, we have not encountered other interventions that show efficacy similar to that of the positive control dexamethasone. In addition, B1R antagonists inhibit development of arthritis and joint destruction in animal models. Targeting B1R, the sCD13 receptor, may provide an effective approach to treating chronic inflammatory diseases such as RA because sCD13 modulates the functions of almost all cell types involved in RA pathogenesis. Thus sCD13 and its receptor provide therapeutic targets for RA, and potentially a broader range of immune-mediated diseases.

## Methods

### Cell culture.

Procedures involving specimens obtained from human subjects were performed under a protocol approved by the University of Michigan Institutional Review Board. Briefly, RA FLSs were harvested from human STs obtained at arthroplasty or synovectomy from RA joints (45, 46). FLSs were then isolated by digestion of human RA STs with a solution containing collagenase type II (650 U/mL), dispase (2.4 U/mL), and DNase (10,000 dornase U/mL). FLSs were maintained in RPMI medium supplemented with 10% FBS and 1% penicillin/streptomycin and were used between passages 3 and 8. Cells were then synchronized in serum starvation medium (RPMI with 0.1% FBS) for 24 hours before addition of appropriate stimuli.

To evaluate the binding of sCD13 to B1R on RA FLSs, we used the bissulfosuccinimidyl suberate (BS3) cross-linker to stabilize the interaction of sCD13 with B1R. RA FLSs were serum-starved for 2 hours followed by incubation with sCD13 in PBS for 1 hour at 4°C before treatment with 2 mM BS3 for 30 minutes. Then, quench solution was added to the reaction mixture for 15 minutes at room temperature. Cells were lysed and proteins were isolated.

To detect B1R on FLSs, human RA FLSs were stimulated with IL-1β (5 ng/mL) or TNF-α (25 ng/mL) for 0.5–24 hours. To assess the phosphorylation status of Erk1/2, RA FLSs were incubated with or without a B1R antagonist (SSR240612, 1 μM) for 1 hour before stimulation with sCD13 (4.8 nM) for 20–25 minutes. The bradykinin agonist DABK (1 μM) was also used to stimulate FLSs with or without B1R antagonist.

### Generation of Cd13^–/–^ mice.

*Cd13^–/–^* mice were generated at the University of Michigan Transgenic Animal Model Core. CRISPR/Cas9 technology was used to introduce a premature termination codon in exon 2, chromosome 7, from position 79841638 to 79842251. CRISPOR (crispr.tefor.net) was used to select 4 single-guide RNAs (sgRNAs) (47). sgRNAs were synthesized by Synthego (48) and then tested for chromosome break formation in mouse zygotes by pronuclear microinjection. Briefly, ribonucleoprotein (RNP) complexes were formed by combination of 30 ng/μL of sgRNA with 30 ng/μL of enhanced-specificity Cas9 protein (Sigma-Aldrich) (49). RNP was microinjected into mouse zygotes that were allowed to develop to blastocyst embryos in vitro. DNA was extracted from individual blastocysts (~64 cells per embryo), and PCR primers were used to amplify a 680 bp genomic DNA fragment including the sgRNA targets (forward primer: 5′-CTCAGGAGAAGAATAGGAATGCAGAGAAC-3′; reverse primer: 5′-CATCCAAGGAACCCTTTCATACCACAA-3′). Amplicons were submitted for Sanger sequencing, and sgRNA C103G3 was selected to produce mutant mice because of its high activity.

Mouse zygotes were obtained by mating of C57BL/6J mice (The Jackson Laboratory, stock 000664). Zygotes were microinjected with RNP based on C103G1 and transferred to pseudopregnant B6D2F1 mice (The Jackson Laboratory, stock 100006). DNA was extracted from tail tip biopsies and amplified with the indel test primers. Five founders showing low–molecular weight bands in addition to WT bands were selected and mated with WT C57BL/6J mice. TOPO TA cloning and sequencing of DNA isolated from obligate heterozygote G1 pups showed the transmission of 6 mutant alleles, including 3 that introduced premature termination codons predicted to result in nonsense-mediated mRNA decay and absence of ANPEP protein (50). The *Cd13^–/–^* mice generated in the transgenic core of the University of Michigan are healthy and breed normally. Confirmation of Cd13 deficiency in these mice was shown by absence of measurable serum sCD13 in *Cd13^–/–^* mice compared with WT (Supplemental Figure 10).

### Generation of B1R^–/–^ mice.

The *B1R^–/–^* mouse strain used for this research project, B6.129P2-Bdkrb1^tm1Dgen^/Mmnc, RRID:MMRRC_011608-UNC, was obtained from the Mutant Mouse Resource and Research Center (MMRRC), an NIH-funded strain repository, and was donated to the MMRRC by Deltagen.

### Arthritic mouse models.

We incorporated several arthritis models in this study to determine the role of the sCD13/B1R axis. Acute inflammation was induced by injection of TNF-α or zymosan into mouse knee joints. We also used the K/BxN serum transfer arthritis model. Since these models are mainly driven by myeloid cells, we also incorporated an antigen-induced arthritis model to determine whether the sCD13/B1R axis also plays a role in a model driven by lymphocytes.

To determine the role of CD13 in acute joint inflammation, *Cd13^–/–^* mouse knees were injected with proinflammatory cytokines. After anesthetizing of WT C57BL/6J (The Jackson Laboratory, stock 000664) and *Cd13^–/–^* mice, TNF-α (14 μM) or PBS was injected into mouse knee joints. Knee joints were measured at day 0 and 24 hours after injection by an observer blinded to the experimental groups (19, 20). Mice were euthanized after 24 hours, and knees were harvested for further analysis. All experiments with mice were performed using both male and female mice between the ages of 10 and 12 weeks.

Zymosan-induced arthritis (ZIA) was induced by intra-articular injection of zymosan (*Saccharomyces cerevisiae*) in WT mice, as follows: 30 mg zymosan was dissolved in 1 mL of PBS. The solution was boiled twice and sonicated. Before the procedure, all mice were anesthetized with ketamine (60 mg/kg) i.p., and mice then received 20 μL/knee of either PBS or zymosan (30 mg/mL) in each knee. Before intra-articular injections, mouse knees were measured for joint circumference with a caliper, as described previously (51). Circumference measurements were taken at 24 and 48 hours for all mice. Each mouse was injected with B1R antagonist (SSR240612 at 0.82 mM) i.p. at 0 and 24 hours as published (52, 53). After euthanasia, knees were harvested for homogenate preparation and cytokine analysis or embedded in OCT compound for cryosectioning. A parallel study was performed in WT and *B1R^–/–^* mice.

We performed K/BxN serum transfer arthritis using *Cd13^–/–^* mice and C57BL/6J WT mice. Arthritis was induced by injection of *Cd13^–/–^* and WT mice i.p. with K/BxN serum (100 μL) on days 0 and 2 (22, 23). These mice were treated with daily i.p. injections of B1R antagonist (SSR240612 at 0.82 mM) starting from day 0 (52–54). Joint circumference of mouse ankles was measured with calipers. The joint circumference at day 0 (before serum injection) was considered background circumference and subtracted from measured circumference at each time point for WT and *Cd13^–/–^* mice. Mice were euthanized on day 8, and the ankles were harvested. We also performed K/BxN serum transfer arthritis in WT and *Cd13^–/–^* mice with daily i.p. injection of B1R antagonist starting from day 0. Some of the ankles were embedded in OCT compound and cryosectioned for immunofluorescence, while others were homogenized and ELISAs were performed for determination of proinflammatory cytokines in these homogenates.

To determine the role of CD13 in an antigen-induced arthritis model, we used the methylated bovine serum albumin (mBSA) model (Figure 6A). Female C57BL/6J mice were immunized on day 0 with an emulsion of 0.25 mg of mBSA (Sigma-Aldrich) in PBS/Freund’s complete adjuvant (FCA) by a subcutaneous injection at the base of the tail. Mice received a 0.1 mg mBSA-FCA booster injection on day 7. On day 21, the mice received either PBS or mBSA (10 μg in PBS) by intra-articular injection into the knee joint. For treatment, 4 experimental groups were included: (a) i.p. injection of B1R antagonist SSR240612 (2 mg/kg) 3 times a week starting from day 0 to day 21, then daily injection of the drug until the end of the study; (b) i.p. injection of vehicle control (2.2 % DMSO/97.8% PBS) 3 times a week starting from day 0 to day 21, then daily injection of the drug until the end of the study; (c) daily i.p. injection of B1R antagonist SSR240612 (2 mg/kg) starting on day 20 until the end of the study; and (d) daily i.p. injection of vehicle control (2.2% DMSO/97.8% PBS) starting on day 20 until the end of the study. Endpoints for this study included joint circumference measurement, cytokine in knee homogenates, immunofluorescent staining in frozen knees, and H&E staining, as well as μCT analysis.

Injection of sCD13 into WT and *B1R^–/–^* mice was performed as previously described (8).

### μCT imaging and morphometric analyses.

Hind limbs were fixed in 10% neutral-buffered formalin for 48 hours and stored in 70% ethanol until imaging. Samples were then rehydrated in PBS 24 hours before imaging, and 4 samples were imaged together in a water-filled sample holder. μCT imaging was performed at 50 kV, 500 μA, with a 0.4° rotation step and 3 frame averages resulting in an 8.9 μm isotropic voxel size. A 0.5 mm aluminum filter was used to reduce beam-hardening artifacts, and a standard hydroxyapatite phantom consisting of water and 200 mg/cm^3^ and 800 mg/cm^3^ standards was used to calibrate images to bone mineral density. Cortical bone erosion was qualitatively scored in a blinded fashion using a 0–3 cortical bone erosion score, as previously reported (55). Separate scores were determined at the femoral proximal joint margin and in epiphyseal cortical bone, and the 2 scores were summed for a composite bone erosion score of the whole joint. Metaphyseal and epiphyseal trabecular bone volumes were manually contoured in a blinded fashion and then quantitatively analyzed in 3D to derive morphometric trabecular parameters, as previously described (56) and per standardized guidelines (57). Metaphyseal and epiphyseal cortical bone volumes were derived automatically by dilation of trabecular volumes and segmenting of background and endocortical tissue. Image contouring was performed in MATLAB (Mathworks Inc.), and bone morphometry parameters were calculated using the ImageJ (NIH) plug-in BoneJ (58), employing the MATLAB-ImageJ interface Miji (59).

### Western blotting.

Equal amounts of protein were separated by SDS-PAGE and electroblotted onto nitrocellulose membranes. The blot was probed with monoclonal anti-B1R antibody followed by stripping and reprobing with WM15, a monoclonal anti-CD13 antibody (BioLegend 301701; clone WM15). In separate experiments the blots were probed for phospho-Erk1/2 (Cell Signaling Technology 4376S; clone 20G11) and total Erk1/2 (Cell Signaling Technology 9102) (22, 60). β-Actin (Sigma-Aldrich A2066) was used as loading control.

### Histology.

To examine whether binding of sCD13 to B1R occurs on FLSs, human RA FLSs were preincubated with sCD13 (96 nM) for 1 hour at room temperature, and subsequently stained with a mouse anti–human B1R antibody (LSBio LC-C196744-100; clone 3A2) (8, 60). Slides were mounted using the Slowfade Gold reagent containing DAPI (Molecular Probes), and images were acquired using an Olympus BX51 confocal microscope (Olympus, Tokyo, Japan). Alternatively, RA STs were embedded in OCT compound and cryosectioned. Immunofluorescence was performed using either anti–human B1R (5.0 μg/mL; LSBio LC-C196744-100; clone 3A2), anti-CD68 (0.73 μg/mL; Cell Signaling Technology 76437T; clone D4B9C), anti–von Willebrand factor (anti-vWF; 10 μg/mL; Thermo Fisher Scientific PA5-16634), or anti-CD55 (4.3 μg/mL; GeneTex GTX113170; clone N1C2) antibody, or purified IgG (Thermo Fisher Scientific 31235). Cryosections were incubated with Alexa Fluor secondary antibodies (10 μg/mL; Vector Laboratories) for 1 hour at 37°C. Sections were mounted with VECTASHIELD antifade mounting medium with DAPI (Vector Laboratories) and visualized under an Olympus microscope (Olympus, Tokyo, Japan) (7, 22). To quantify the number of B1R-positive cells in the tissues, B1R- and cell marker–positive cells in patients were counted by 2 blinded laboratory members.

To detect the presence of MNs/macrophages in mouse models of inflammatory arthritis, immunofluorescence on arthritic joints was performed using rat anti–mouse F4/80 (Thermo Fisher Scientific MA1-91124; clone CI:A3-1) and Alexa Fluor 488–tagged (green, Molecular Probes) secondary antibody. Quantification of F4/80-positive cells was done by ImageJ.

### Protein structure prediction.

The protein structure of monomeric B1R and sCD13 can be predicted by C-I-TASSER (61), the latest version of the I-TASSER structure prediction algorithm with newly added sequence-derived contact potential (62, 63). To generate the structure complex between B1R and CD13, dimeric templates identified by SPRING (64) and COTH (65) were combined by TACOS (66), an extension of C-I-TASSER for dimer structure modeling, to generate an initial dimer structure. The monomeric C-I-TASSER models of B1R and CD13 were then superposed by template modeling score (67) to the TACOS initial dimer structure, followed by ModRefiner (68) full atomic refinement, to obtain the final complex model.

### Quantitative reverse transcription PCR to detect B1R.

mRNAs from RA FLSs were extracted followed by cDNA synthesis. Gene expression was measured using a 7500 Quantitative PCR System (Applied Biosystems) and 2× TaqMan Universal PCR Master Mix (Thermo Fisher Scientific) according to the manufacturer’s instructions (22). In separate experiments, the ViiA 7 system was used along with Power SYBR Green Master Mix (Thermo Fisher Scientific).

### MN chemotaxis using sCD13 and DABK.

Peripheral blood was collected in heparinized tubes from healthy adult donors. MNs were harvested using Accu-Prep and Percoll gradient methods as previously described (7, 60) and were more than 95% pure, and viability was greater than 98% by trypan blue exclusion. They were then preincubated with either SSR240612 or R715 for 30 minutes, then subjected to migration assays in modified Boyden chambers. sCD13 (4.8 nM) and des-Arg9-bradykinin (DABK; 1 μM) were used as stimuli. The concentration of DABK was based on our dose-response curve performed with DABK (data not shown) and published data (69, 70). Three high-power fields (×400) in each well were counted by an observer blinded to the experimental groups.

### Ex vivo culture of RA ST.

STs received from arthroplasty and synovectomy procedures were dissected into small pieces of about 1–2 mm^3^ as previously described (8, 21). Ex vivo cultures contained a representative mixture of the various cell populations present in RA ST in vivo. Each piece of RA ST was incubated in 1 mL Connaught Medical Research Laboratories (CMRL) medium (Invitrogen) containing 10% FBS for 24 hours, then incubated for an additional 48 hours in fresh medium with or without SSR240612 (a B1R antagonist; 50 μM), or dexamethasone (25.5 μM) as a positive control for suppression (8, 21). ELISAs were performed on culture supernatants from the 0- to 24-hour and 24- to 72-hour incubation periods to measure secretion of MCP-1/CCL2 and IL-6. The ratio of secreted protein at 72 hours to the amount secreted at 24 hours was used to determine fold change in secretion for each ST piece. We used 6–12 replicates of ST from each of 3 different RA patients.

### Cytokine measurement.

Cytokines in culture media or joint homogenates were measured using ELISA kits from Invitrogen, R&D Systems, and Aviva Systems Biology, as well as the LEGENDplex kit from BioLegend.

### Single-cell transcriptome analysis.

We extracted published single-cell RNA sequencing (RNA-Seq) data sets generated from RA synovium from 27 patients (71). Single-cell transcriptome data sets were read using Seurat v4 (72), and analyzed as described previously (71). In brief, high-quality transcripts were used to create a Seurat object and then were normalized. Standard workflow from Seurat was used to find the relevant components with principal component analysis (PCA) (npcs = 30) and to visualize the results with uniform manifold approximation and projection (UMAP) (reduction = “pca”; dimensions = 1:20). The FeaturePlot, VlnPlot, and DoHeatmap functions of Seurat were applied to generate the feature plots, violin plots, or heatmaps for genes of interest such as *ANPEP*, *MMP* genes, and *BDKRB1*.

### Statistics.

Results are expressed as mean ± SD. Normality test was conducted to determine whether the data were normally distributed or skewed. Normally distributed data were analyzed by 2-tailed Student’s *t* test, 1-way ANOVA, or 2-way ANOVA, while skewed data were analyzed by Mann-Whitney test or Kruskal-Wallis test. Data analysis was done with GraphPad Prism software version 8. A *P* value less than 0.05 was considered significant.

### Study approval.

All animal protocols were approved by the Institutional Animal Care and Use Committee at the University of Michigan. This study was approved by the University of Michigan Institutional Review Board. Participants gave written informed consent before being included in the study.

## Author contributions

CL, PST, MGR, SM, PLC, QW, ENM, MEL, SV, MNM, WDB, JLH, SO, CIA, MA, GH, MMO, and MAA designed and executed various aspects of the study, including Western blotting, immunofluorescence, ELISA, flow cytometry, MN chemotaxis, homogenizing of mouse joints, and data analysis. CL, PST, MGR, SM, PLC, MAA, and JLH helped in performing animal models of arthritis. PST, MGR, PLC, JHR, CL, and MAA designed animal models of arthritis. AMR and AJA helped in designing and interpreting the results. CZ developed the structure model of the CD13-B1R complex. AGU, JNL, and KCC provided RA ST to perform in vitro assays with RA FLSs and ex vivo organ culture model. TM, RP, and LJ executed μCT and performed the analysis. TLS generated Cd13-null mice. VEG, MAA, and DAF were instrumental in designing and developing this project, and writing the manuscript. PST and CL are co–first authors. PST is listed first because she organized the data for development of the manuscript in addition to planning and leading the experiments for the revision.

## Supplementary Material

Supplemental data

## Version 1 04/19/2022

In-Press Preview

## Version 2 06/01/2022

Electronic publication
