# Supplementary material for: Soluble CD13 induces inflammatory arthritis by activating the bradykinin receptor B1
Source: J Clin Invest. 2022 Jun 1;132(11):e151827. doi: 10.1172/JCI151827 (PMC9151693; doi:10.1172/JCI151827)
Supplement: Supplemental data [file jci-132-151827-s068.pdf]

Supplemental Figure 1. Flow cytometry was performed using Alexa Fluor 647-conjugated anti-human B1R to confirm B1R expression on RA FLS. This assay was repeated with FLS from 2 RA patients. We found that more than 80% of cells express B1R, suggesting that abundant B1R is available on the FLS surface in RA.

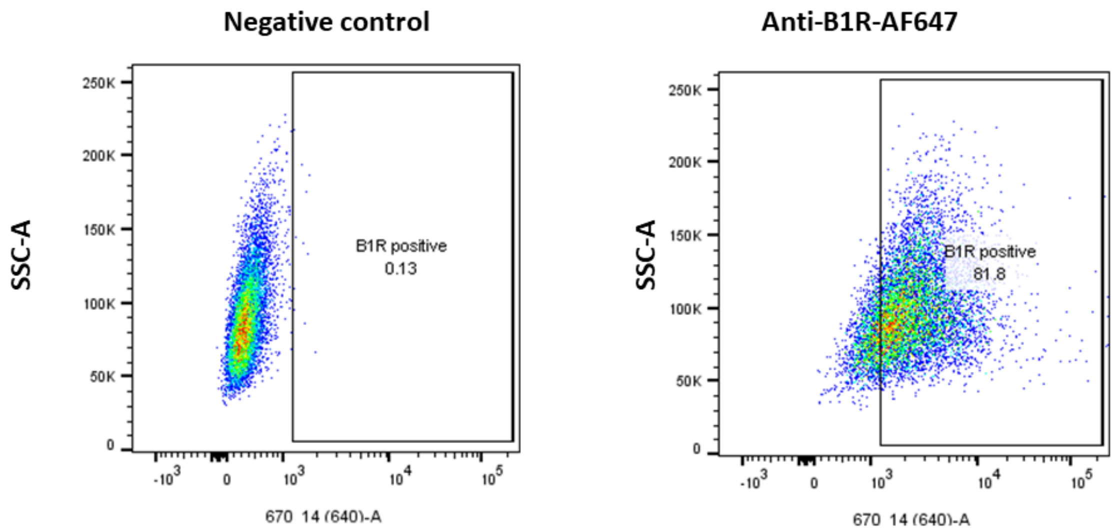

Supplemental Figure 2. B1R (green) is expressed on CD90/Thy1 (red) positive RA SFs. Nuclear staining is done by DAPI (blue). Representative pictures of 3 RA patients. Original magnification, ×200-×400

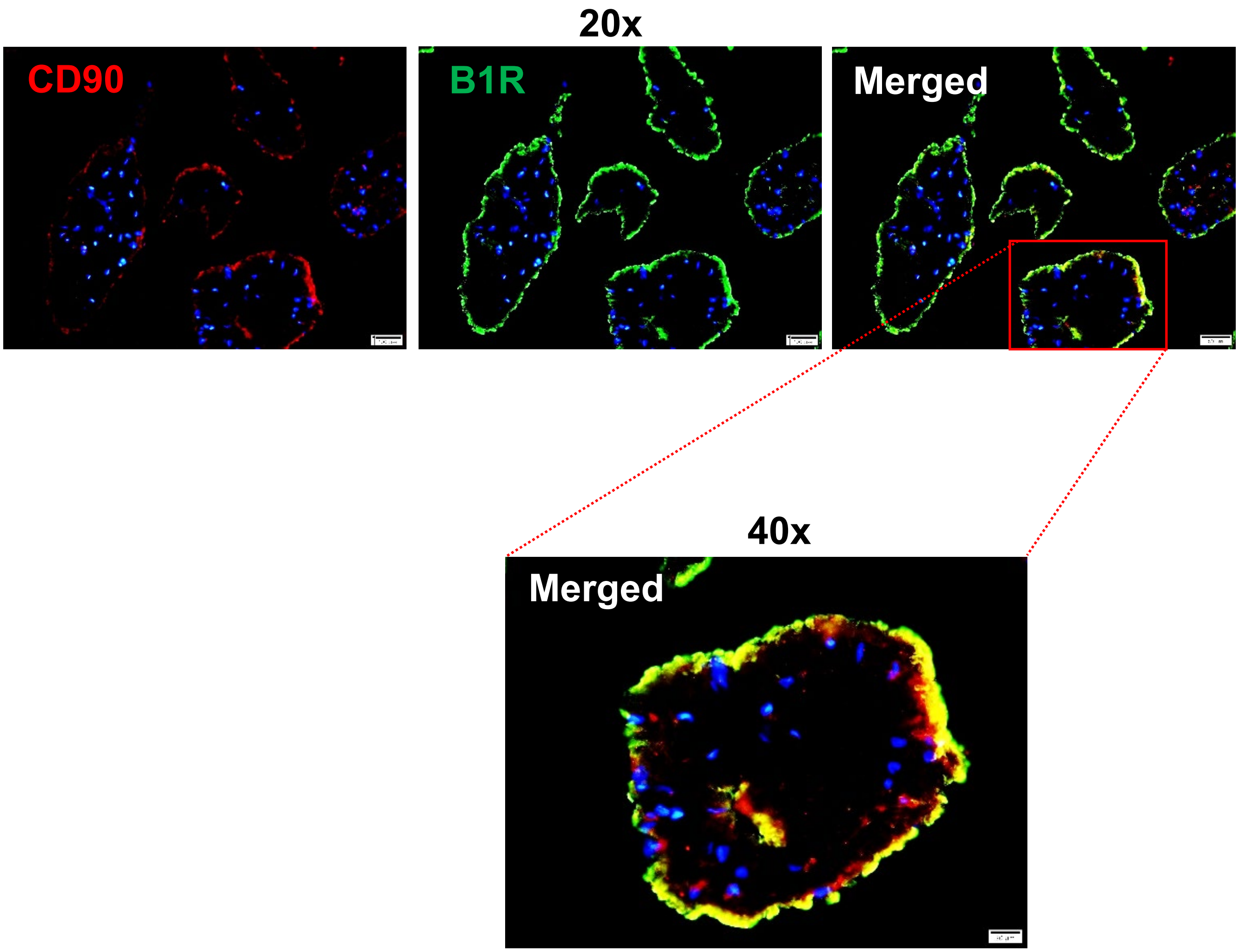

Supplemental Figure 3. Single cell-RNA seq analysis of published data from Zhang et al (1). A total of 13 clusters including 5 fibroblast subsets, 2 monocyte subsets, and other immune cells were identified. UMAPs of *ANPEP*, *BDKRB1*, and *MMP14* are presented here.

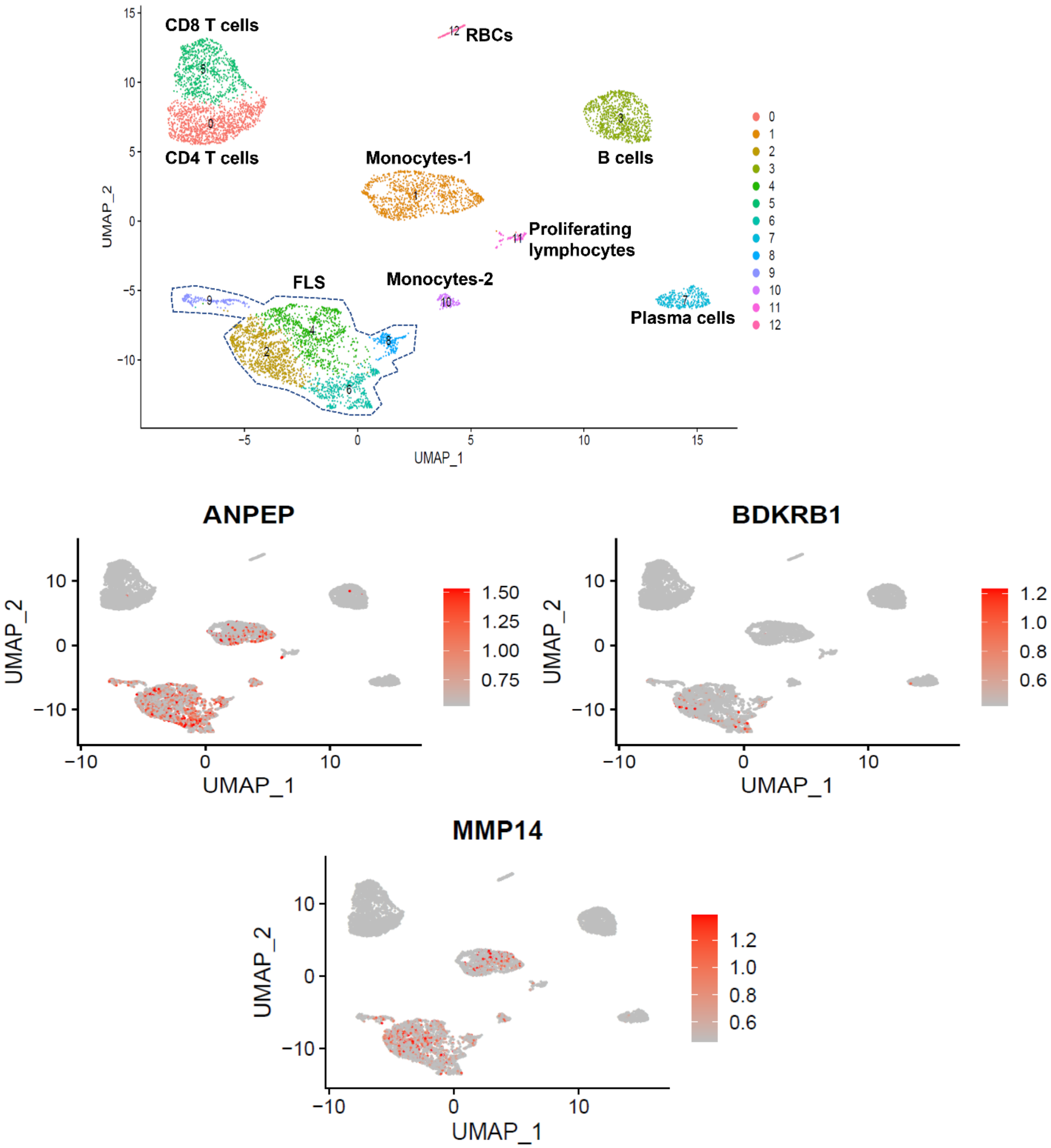

1. Zhang F, Wei K, Slowikowski K, Fonseka CY, Rao DA, Kelly S, et al. Defining inflammatory cell states in rheumatoid arthritis joint synovial tissues by integrating single-cell transcriptomics and mass cytometry. *Nat Immunol.* 2019;20(7):928-42.

Supplemental Figure 4. Both sCD13 and DABK induced Erk1/2 phosphorylation in RA FLS, which was reduced by SSR240612. Representative blots of 4 RA patients. NS: Non-stimulated.

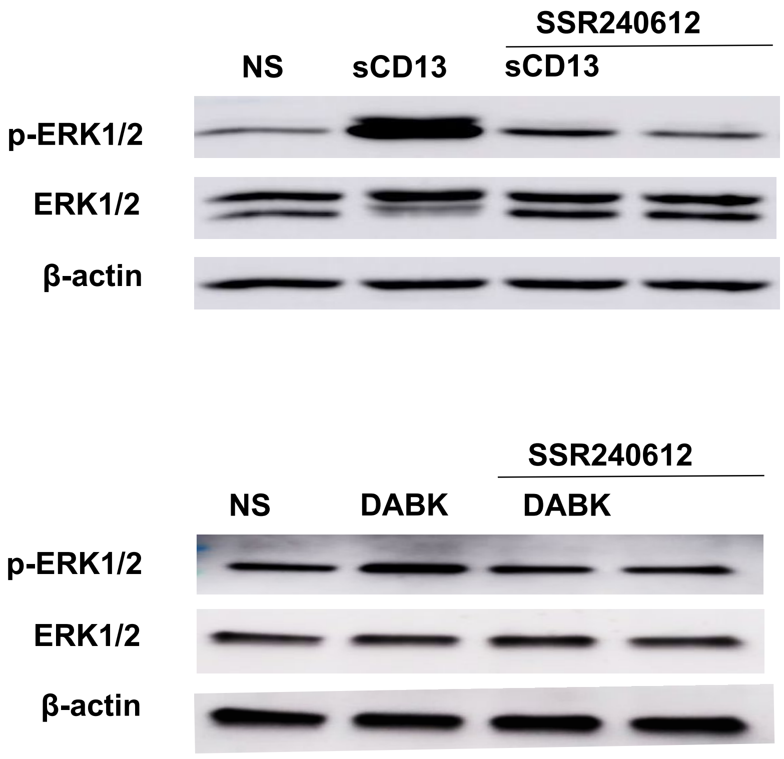

Supplemental Figure 5. Knockdown of *BDKRB1* in RA FLS was confirmed by quantitative PCR. The experiment was conducted in 3 RA patient lines. *BDKRB1* was knocked down approximately 93% with the B1R siRNA compared to control siRNA. Results are expressed as mean ± SD.

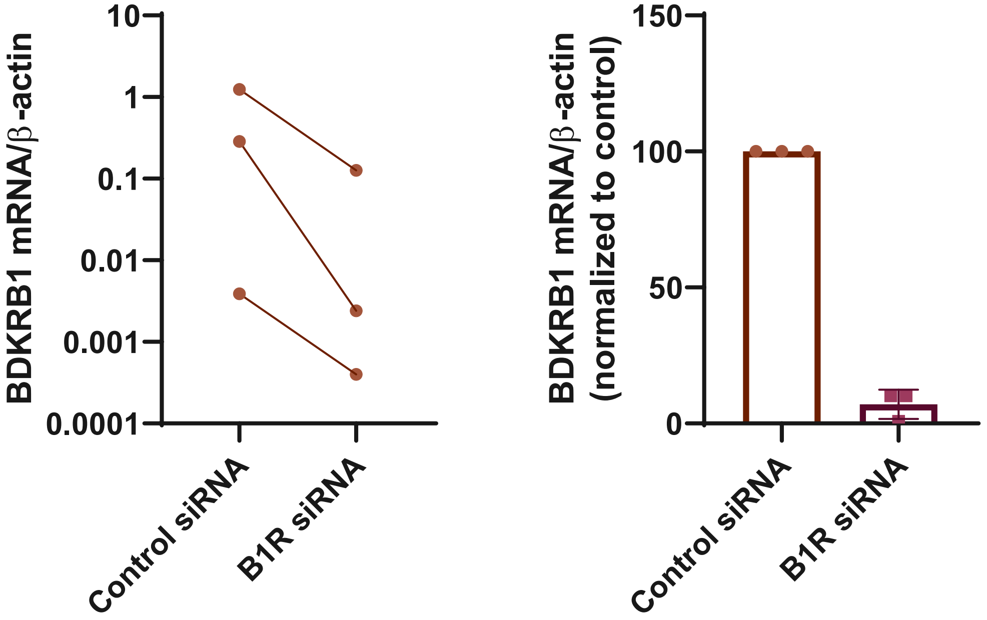

Supplemental Figure 6. In WT mice, sCD13 levels in joint homogenates were significantly elevated in zymosan-injected knees compared to PBS controls. Results are expressed as mean ± SD. *p<0.05 was considered significant. Significance was determined by Mann Whitney U test.

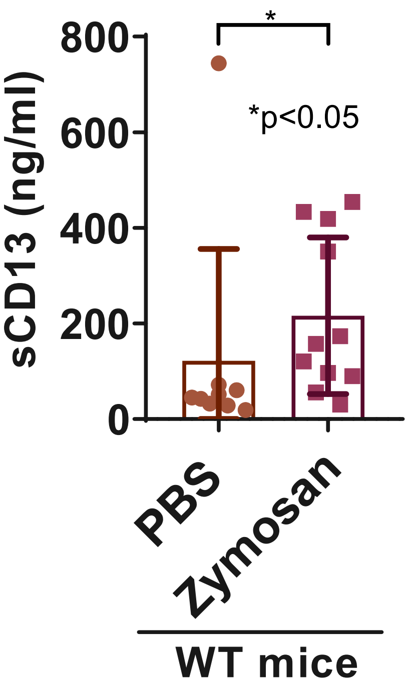

Supplemental Figure 7. Effect of B1R blockade in WT and *Cd13$^{-/-}$* mice in the K/BxN serum transfer arthritis model. (**A**) Arthritis in *Cd13$^{-/-}$* mice (n=10) was less pronounced compared to WT mice (n=9-13) as measured by joint circumference, with significant difference observed on day 7; (**B**) There was no difference in arthritis between SSR240612-treated *Cd13$^{-/-}$* mice (n=6) and SSR-treated WT mice (n=10) except for day 8; Results are expressed as mean ± SD. *$p<0.05$ was considered significant. Significance was determined by two-way ANOVA. SSR: SSR240612

A

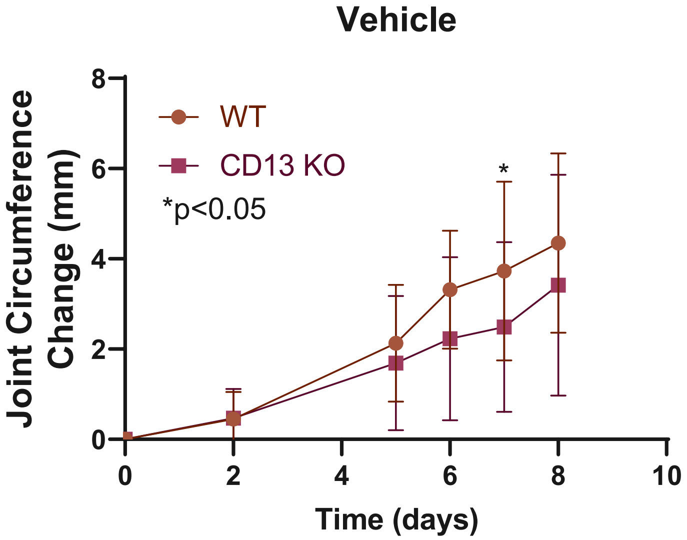

B

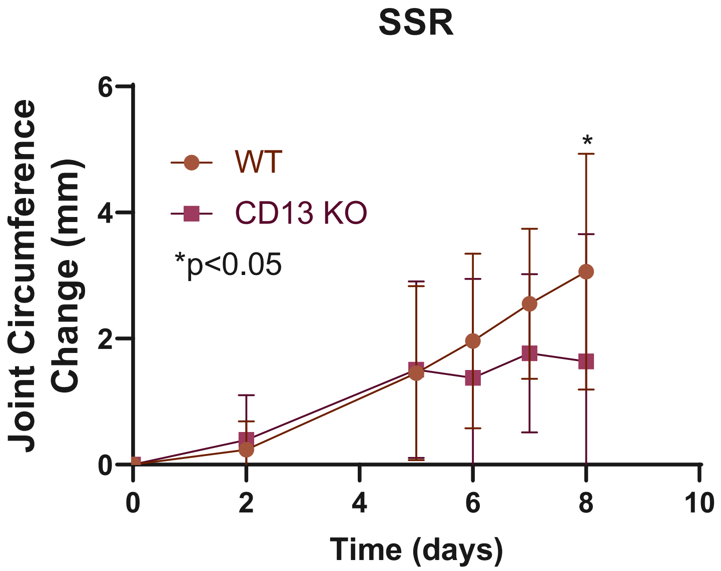

Supplemental Figure 8. Representative H&E staining of murine knee joints from the mBSA antigen-induced arthritis model (A). Tissues were scored for the degree of inflammatory cell infiltration and structural damage by two blinded observers (B). Results are expressed as mean ± SD and *p<0.05 was considered significant. SSR: SSR240612

A

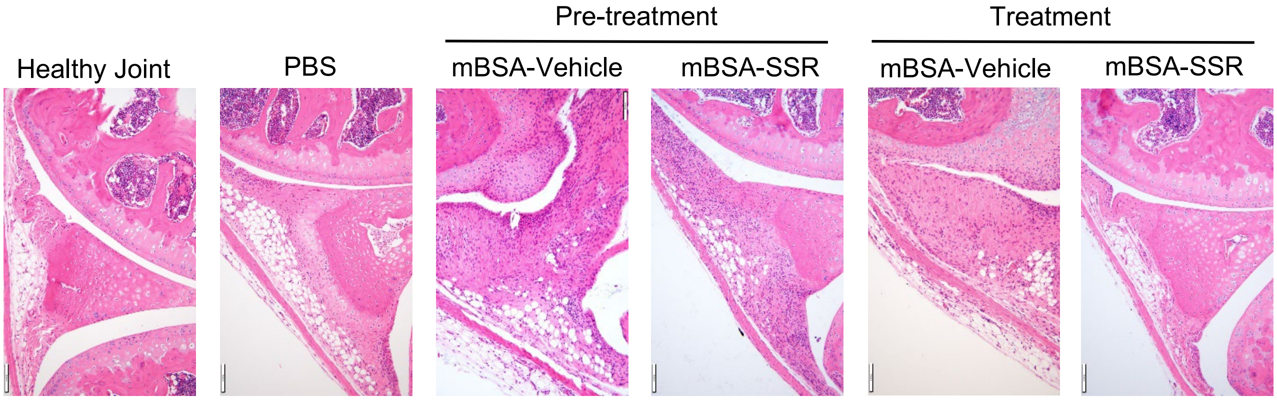

B

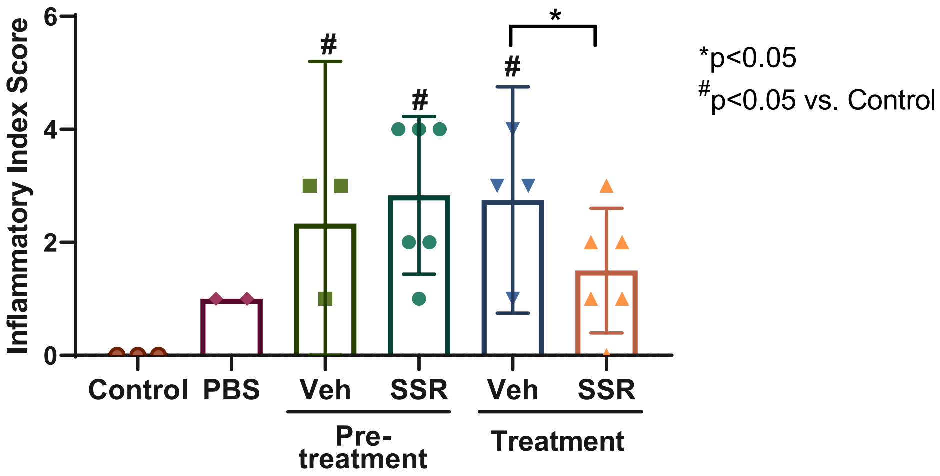

Supplemental Figure 9. MicroCT analysis of femoral trabecular bone in (A) epiphyseal and (B) metaphyseal regions from the mBSA antigen-induced arthritis model. Results are expressed as mean ± SD and *p<0.05 was considered significant. Significance was determined one-way ANOVA. SSR: SSR240612

A

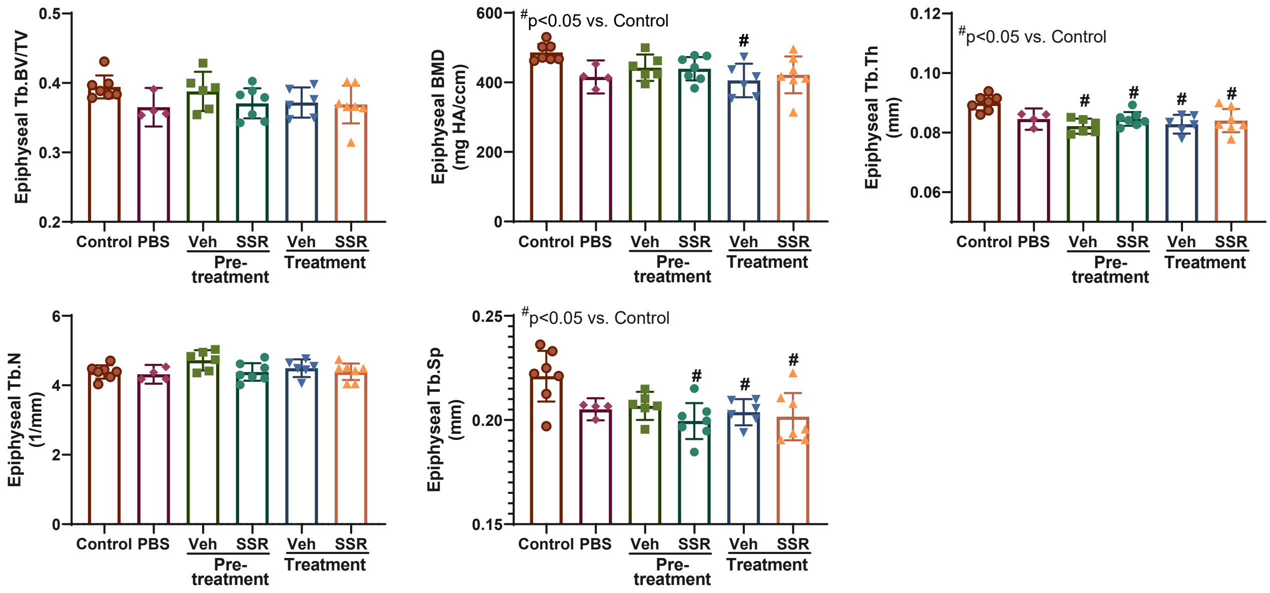

B

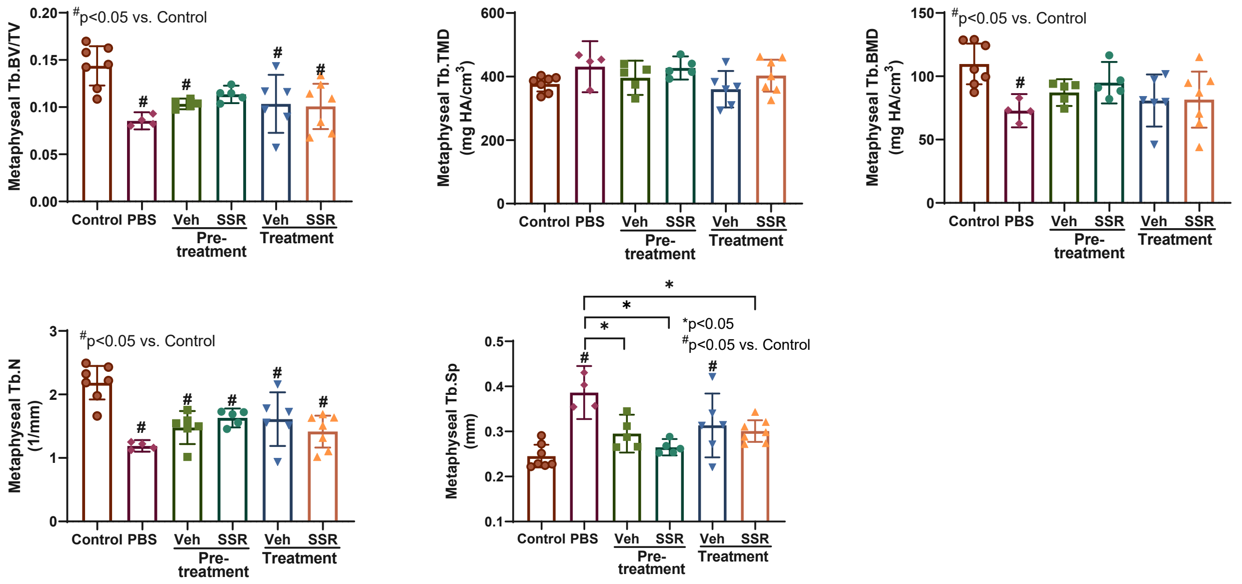

Supplemental Figure 10. CD13 ELISA showing the absence of sCD13 in serum of *Cd13*$^{-/-}$ mice compared with the measurable sCD13 in wildtypes.

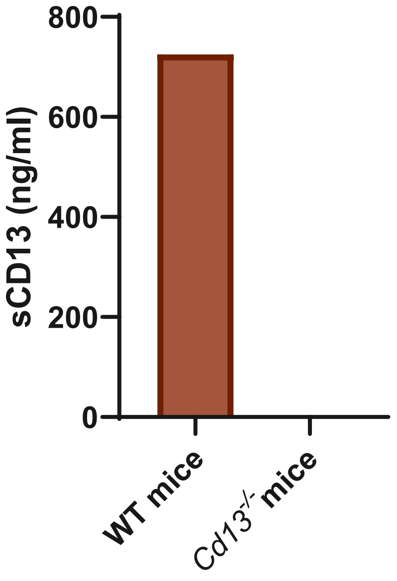

Supplemental Table 1. C-I-TASSER program was used to predict the protein structure of monomeric B1R and CD13, as shown in Figure 2C. The contact amino acids of B1R and CD13 are listed in the table.

The contact amino acids of B1R and CD13

B1R	CD13	Distance (Å)	B1R	CD13	Distance (Å)
5	318	7.746900993	277	293	6.325686208
25	318	7.438206908	6	315	6.965624739
280	335	6.869577352	278	298	3.42842194
279	297	5.585056222	286	295	6.212120491
26	295	7.259907231	273	295	6.964145389
23	315	7.400151958	4	315	6.360813549
283	329	7.535117451	6	290	7.503215711
275	296	7.396998445	4	310	7.156718452
6	292	5.624852798	282	294	3.40733004
5	292	6.70569206	25	292	5.265314141
6	318	6.381793008	282	329	7.292917455
278	297	1.673620626	274	297	6.449086524
283	323	7.037282856	4	312	4.733710278
277	296	3.911660773	286	326	7.718066662
7	292	6.27211328	279	296	4.9636957
283	295	6.084130916	283	327	7.921580713
278	295	7.670945053	4	290	6.340856488

Supplemental Table 2. Cytokine production in joints after PBS or Zymosan injection in wild type and B1R knockout mice. Cytokine levels were measured using the BioLegend's LEGENDplex™ assay kit, and were normalized to the weight of the joints. Data are presented as mean ± SEM, with $p<0.05$ considered as statistically significant (bolded). Significance was determined by Kruskal-Wallis test or one-way ANOVA.

Cytokines (mean ± SEM)	Wild type mice			B1R knockout mice			Wild type vs. B1R mice	
	PBS (n=9)	Zymosan (n=12)	p-value (PBS vs. Zymosan)	PBS (n=9)	Zymosan (n=12)	p-value (PBS vs. Zymosan)	p-value (PBS vs. PBS)	p-value (Zymosan vs. Zymosan)
IL-1β (pg/ml)	3.4 ± 1.0	24.2 ± 3.7	**0.0008**	16.5 ± 5.0	33.3 ± 3.2	**0.008**	0.08	0.22
IL-6 (pg/ml)	4.6 ± 1.4	138.7 ± 33.2	**0.0002**	15.0 ± 4.0	157.3 ± 47.9	**0.02**	0.85	0.99
MCP-1/CCL2 (pg/ml)	25.8 ± 8.3	43.3 ± 5.0	0.07	24.5 ± 4.5	54.1 ± 6.4	**0.005**	0.99	0.99
IL-27 (pg/ml)	71.3 ± 17.7	99.9 ± 21.2	0.99	132.5 ± 51.3	198.1 ± 28.9	0.06	0.28	**0.01**
IFN-γ (pg/ml)	2.3 ± 0.5	5.0 ± 2.1	0.99	3.9 ± 1.0	11.6 ± 3.8	**0.03**	0.99	**0.04**
TNF-α (pg/ml)	1.3 ± 0.3	16.7 ± 2.8	**0.0004**	3.3 ± 0.9	16.7 ± 3.3	**0.0005**	0.98	0.96
IFN-β (pg/ml)	5.0 ± 0.2	5.1 ± 0.2	0.99	12.6 ± 5.4	19.5 ± 4.2	0.18	0.99	0.12
IL-1α (pg/ml)	111.9 ± 28.9	271.0 ± 51.9	**0.007**	567.5 ± 412.1	169.6 ± 13.6	0.99	0.35	0.54
IL-17A (pg/ml)	1.4 ± 0.8	3.1 ± 1.1	0.99	6.5 ± 3.3	9.5 ± 1.4	0.10	0.99	**0.02**
IL-10 (pg/ml)	6.8 ± 0.6	11.6 ± 3.1	0.99	16.6 ± 5.4	27.4 ± 2.8	0.053	0.89	**0.008**
IL-23 (pg/ml)	125.3 ± 22.0	255.9 ± 53.6	0.51	316.2 ± 104.0	464.5 ± 59.0	0.39	0.22	0.07
